# Single-Cell RNA Sequencing of Human Lamina Cribrosa Fibroblasts in Glaucoma

**DOI:** 10.3390/ijms27219892

**Published:** 2026-11-04

**Authors:** Caoimhe Normile, Mustapha Irnaten, Oisin Cappa, Vadim Zhernovkov, Jeffrey O’Callaghan, Bruce Moran, Eoghan Culligan, Sherri Feris, Stacy Curry, Abbot F. Clark, David Simpson, Colm O’Brien

**Affiliations:** 1 UCD Clinical Research Centre, Mater Misericordiae University Hospital, D07 KX5K Dublin, Ireland; 2 Department of Ophthalmology, Mater Misericordiae University Hospital, D07 R2WY Dublin, Ireland; 3 The Wellcome–Wolfson Institute for Experimental Medicine, School of Medicine, Dentistry and Biomedical Sciences, Queen’s University Belfast, Belfast BT9 7BL, UK; 4 Systems Biology Ireland, School of Medicine, University College Dublin, D04 V1W8 Dublin, Ireland; 5 Department of Genetics, Trinity College Dublin, D02 PN40 Dublin, Ireland; 6 St Vincent’s University Hospital, D04 T6F4 Dublin, Ireland; 7 Department of Pharmacology & Neuroscience and the North Texas Eye Research Institute, University of North Texas Health Science Center, Fort Worth, TX 76107, USAabe.clark@unthsc.edu (A.F.C.)

**Keywords:** scRNA-seq, glaucoma, lamina cribrosa, ISR, fibrosis

## Abstract

The molecular mechanisms that underlie the extracellular matrix (ECM) remodelling of the human lamina cribrosa (LC) in glaucoma remain obscure. This is the first study to characterise human glaucomatous LC cells using single-cell RNA sequencing (scRNA-seq). Our aim is to characterise transcriptional changes in human LC cells from glaucomatous and normal eyes and identify signalling pathways contributing to optic nerve head remodelling. scRNA-seq was performed on LC cells from glaucomatous and normal donor eyes. Differential gene expression was analysed using Seurat. Cell identity was assessed using SingleR and GTEx reference datasets. Pathway enrichment analysis was performed using Gene Set Enrichment Analysis (GSEA), and transcription factor (TF) motif enrichment was evaluated using i-cisTarget. Validation was performed using quantitative PCR, Western blotting, immunohistochemistry, and immunofluorescence. LC cells were treated with ISRIB and 4-PBA. Analysis of 6131 LC cells using shared nearest-neighbour clustering and UMAP visualisation identified four broad transcriptional groups, which were used for subsequent transcriptional characterisation. Cell type mapping identified a fibroblast/mesenchymal cell origin. Glaucomatous LC fibroblasts demonstrated a pro-fibrotic transcriptional profile, with overexpression of multiple pro-fibrotic genes, master TF regulons involved in cell fate decisions, and TF motifs including SRF, as well as key integrated stress response (ISR) genes such as ATF4, ATF3 and CREB3L1. Enriched pathways included E2F, G2M, the Hallmark EMT gene set and MYC, while the p53 pathway was downregulated. Pharmacologic treatment with ISRIB or 4-PBA reduced expression of stress-response and ECM genes, while ISRIB attenuated the increased cellular metabolic activity observed in glaucomatous LC cells. Bioinformatic analysis of scRNA-seq of human glaucoma LC cells identified increased expression of myofibroblast genes, an EMT-associated/profibrotic transcriptional programme, and cell cycle signalling pathways, TF regulatory elements and stress response genes.

## 1. Introduction

Glaucoma is an age-dependent disease with a prevalence of 2–3% in those over the age of 40 [1]. It is the most common cause of irreversible visual impairment in this age group. It can be defined as a slowly progressive optic neuropathy, with visual field loss due to damage to retinal ganglion cells (RGC) and their axons as they exit the eye at the level of the lamina cribrosa (LC) in the optic nerve head (ONH) [2]. The majority of glaucoma patients have raised intraocular pressure (IOP) though ONH damage is also seen in patients with normal IOP (normal tension glaucoma), while some patients with raised IOP exhibit no optic nerve damage (ocular hypertension). This suggests that IOP is not the only factor damaging the ONH. Elevated IOP occurs due to extracellular matrix (ECM) accumulation and connective tissue (CT) remodelling in the major aqueous humour outflow pathway (trabecular meshwork [TM] and Schlemm’s canal) causing an increase in TM outflow resistance and stiffness resulting in ocular hypertension [3,4,5]. Currently, IOP reduction is the sole recognised treatment.

Much like the TM, the LC is a fenestrated, sieve-like CT within the optic nerve head 6. It is a multi-layered network of load-bearing beams that provide structural support and nutrient supply to the unmyelinated RGC axons in the ONH [6]. Changes in morphology of the LC in glaucoma include compression of CT layers, thinning, posteriorisation of the LC insertion into the scleral canal with excessive ECM deposition and remodelling in the LC region causing fibrosis, and increased levels of the pro-fibrotic growth factor transforming growth factor beta 2 (TGFβ2) [7,8]. These structural alterations, coupled with axon loss, are detected clinically as cupping of the ONH, but the mechanisms driving this process remain incompletely understood.

In recent years, several genome wide association studies (GWAS) have identified over 300 independent risk loci for glaucoma across different ancestries [9,10,11,12]. Bioinformatic gene enrichment pathways have identified ECM organisation, collagen biology, transforming growth factor beta, and vascular development as key disease mechanisms [11,12,13]. Integration of these GWAS studies data with scRNA-seq profiles of ocular cell types has identified fibroblast populations in both the anterior and posterior segments as enriched for glaucoma-associated genetic risk signals, suggesting these cells as likely mediators of disease susceptibility.

LC cells have been shown to have some fibroblast characteristics in glaucoma including the expression of *COL1A1*, *FN1*, and the contractile myo-protein α-SMA (encoded by *ACTA2*) along with increased levels of ECM proteins [14,15,16,17,18]. However, they have not previously been studied at a single cell level. Traditional methods compare the average gene expression of all the cells within a sample, but overlook heterogeneity between individual cells. Single cell RNA sequencing (scRNA-seq) profiles the gene expression of individual cells and has enabled the characterisation of subpopulations of fibroblasts in idiopathic pulmonary fibrosis (IPF) and interstitial lung disease [19,20]. In addition, scRNA-seq in cancer-associated fibroblasts (CAF) has demonstrated “myofibroblastic” and “inflammatory” subpopulations of CAF, and shown that the “inflammatory” subpopulation is also present in normal tissue, suggesting they are recruited during tumour development [21]. Myofibroblasts were first described by Gabbiani in 1971, as cells that combine the morphological features of tissue fibroblasts and contractile smooth muscle cells [22,23]. They play a key role in both normal wound healing and in tissue fibrotic scar formation [24]. Recent scRNA-seq of normal donor TM tissues has identified significant fibroblast and myofibroblast sub-populations [25]. scRNA-seq of the human ONH has identified several different clusters of astrocytes, fibroblasts, vascular endothelial cells, and mural cells [26]. Our study compared patterns of gene expression between groups of LC cells, as well as between normal and glaucomatous LC cells, to identify underlying pathophysiological mechanisms. Unbiased pathway analysis identified multiple altered biological programs, including extracellular matrix, cell cycle, and stress-response pathways. Within this broader context, the integrated stress response (ISR) was selected for further evaluation as a candidate pathway based on prior literature implicating ISR-related signalling in glaucoma models, including studies in trabecular meshwork cells from Zode et al. [27,28]. We therefore examined ISR-associated components in LC cells and assessed the effects of pharmacologic modulation of this pathway, with findings interpreted as part of a broader network of disease-associated transcriptional changes.

## 2. Results

### 2.1. Analysis of Transcriptional Groups

#### 2.1.1. Individual Group Analysis

**Figure 1:**
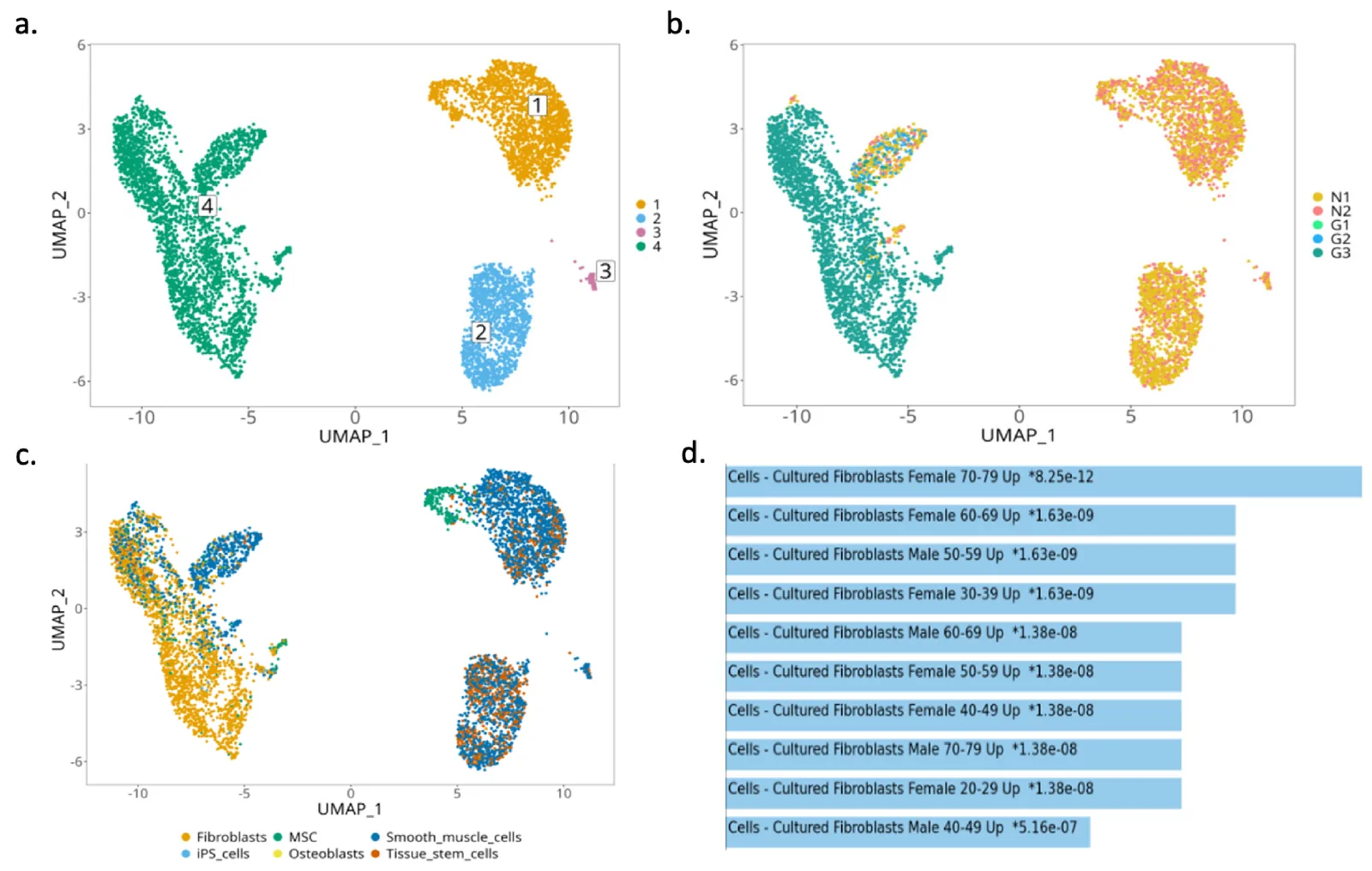
LC cells form four main groups. (**a**) UMAP diagram of all LC cells from normal and glaucoma human donors showing the four distinct groups referred to throughout the article. (**b**) UMAP diagram showing normal and glaucoma donor identity of individual LC cells. Three of the four groups are composed exclusively of cells from normal donors. All glaucoma derived cells were located in Group 4, which also contained cells from normal donors. (**c**) UMAP diagram of cell characterisation using SingleR, colours representing varying cell identities showing that the majority of cells are fibroblasts and are of mesenchymal stem cell origin. (**d**) Graph of cell identity created using GTEx database, highlighting that LC cells most resembled all GTEx cultured fibroblasts. * *p* < 0.05.

Unsupervised clustering of all LC cells (3461 normal cells and 2670 glaucoma cells), followed by UMAP analysis, identified four broad transcriptional groups (Groups 1–4) (Figure 1a). These groups were manually assigned based on distinct populations in UMAP space and represent broad transcriptional populations rather than fine-resolution computational clusters. The distribution of cells across the four groups from normal and glaucoma donors is shown in Figure 1b, with all glaucoma cells located in Group 4. Groups 1, 2 and 3 contained cells from both normal donors (N1 and N2), while Group 4 contained cells from all five donors, including 12.99% of cells from N1 and 7.34% from N2.

Cell-cycle regression was performed to assess the contribution of cell-cycle-associated transcriptional variation to the observed group structure. Following regression, the four broad transcriptional groups remained identifiable (Supplementary Material S1). Quantitative comparison of group assignments before and after regression demonstrated that 98.57% of cells retained their corresponding group assignment, supporting the robustness of these broad transcriptional groups to cell-cycle-associated variation. The corresponding group-assignment concordance matrix is provided in Supplementary Material S2.

#### 2.1.2. Characterisation of the LC Cells

The initial description of LC cells by Hernandez et al. proposed that they were “fibroblastoid” by virtue of their flat polygonal appearance and by their synthesis of Collagens type III and IV, fibronectin and elastin [15]. To verify this, we used SingleR, which showed that the cells were of mesenchymal cell origin (Figure 1c), and the majority, especially the glaucoma cells, had a gene signature similar to that of fibroblasts. Furthermore, we compared the gene expression profile of our LC cells to multiple cell and tissue expression signature datasets using GTEx, which confirmed that the gene signature of LC cells is most similar to fibroblasts (Figure 1d) [29,30].

**Figure 2:**
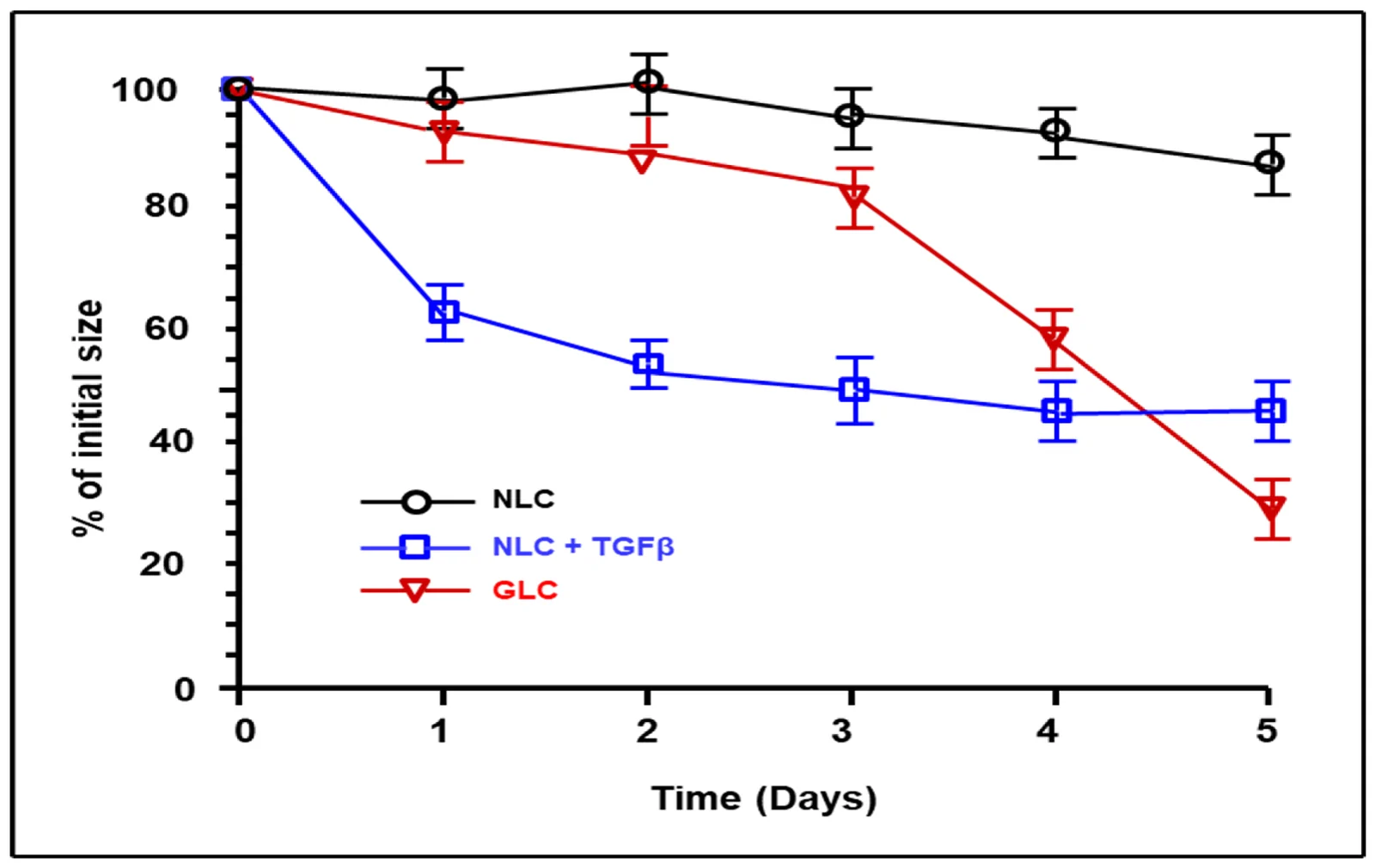
Gel contraction by percentage change relative to initial matrix size, of normal (NLC), normal pretreated with TGFβ (NLC + TGFβ) and glaucomatous LC cells (GLC).

For functional validation of the myofibroblast phenotype of LC cells, we performed gel contraction assays. Pre-treatment of normal LC cells with TGF-β1 significantly increased collagen gel contraction within 24 h compared with untreated controls. Glaucomatous LC cells also demonstrated increased contractile activity, with significant gel contraction observed after 72 h of incubation (Figure 2).

#### 2.1.3. Gene Expression Analysis of Groups

**Figure 3:**
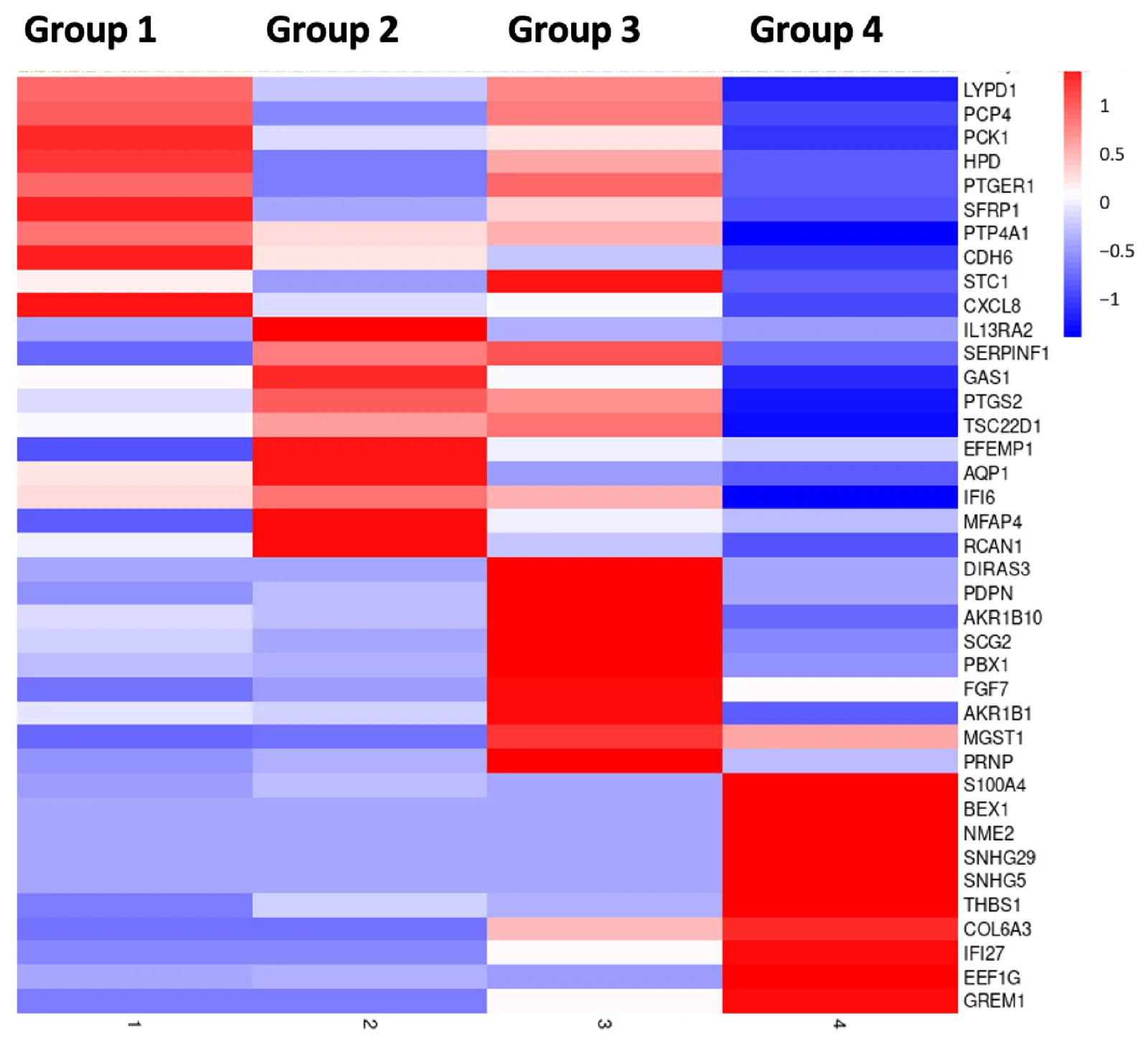
**Heatmap of genes differentially expressed across 4 groups**. Top ten genes most preferentially expressed in each of the four groups. The differential expression highlights the greater separation of Group 4 from all other groups, characterised by relative overexpression of genes including *S100A4*, *THBS1* and *COL6A3*.

Amongst the most highly expressed genes across all four groups were the ferritin genes (*FTL*, *FTH1*), actin-related genes (*TMSB4X* and *ACTB*), the mitochondrial cytochrome oxidase genes (*MT-CO1* and *MT-CO2*), and ribosomal genes (*RPS* and *RPL*) (Supplementary Material S3). Differential expression testing to identify the most significantly enriched marker genes in each of the four groups highlighted 1144 genes (Supplementary Material S4). Each group demonstrated characteristic gene expression, and Figure 3 shows a heat map of the top marker genes for each group. Each group demonstrated a characteristic transcriptional profile, representing distinct transcriptional states/subpopulations within the cultured LC cell dataset.

#### 2.1.4. Group Sub-Classification

**Figure 4:**
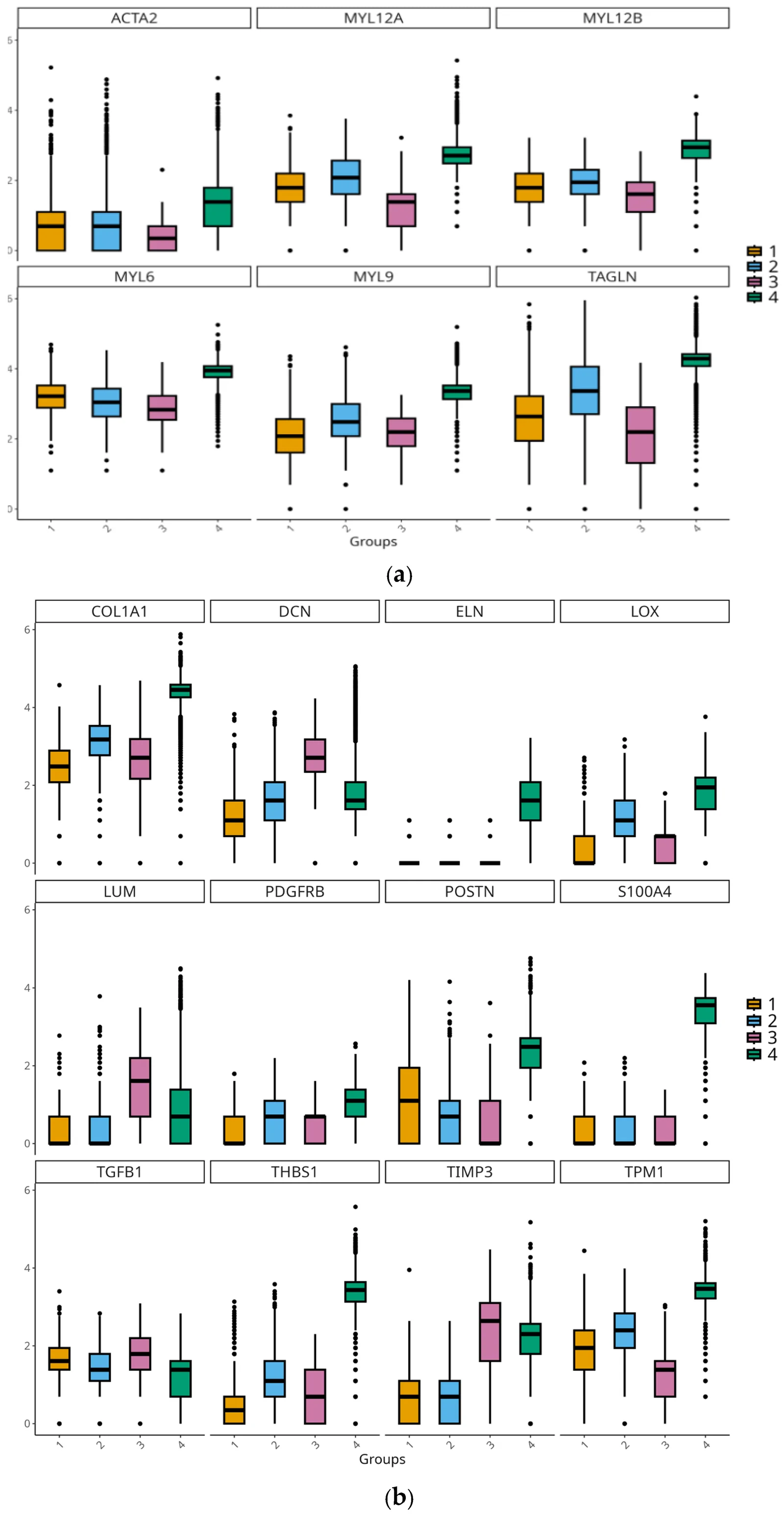
**Expression of genes involved in contractility** (**a**)**, fibrosis and connective tissue formation** (**b**) **across all 4 groups**. Boxplot of contractility, pro-fibrotic and CT-related gene expression across groups 1–4, showing increased expression in group 4.

The hallmark of the activated fibroblast (the “myofibroblast”) is the over-production of contractile myoproteins such as αSMA (*ACTA2*), which can lead to excessive collagen secretion and contraction, resulting in stiff fibrotic scar tissue [31]. *ACTA2* is significantly more highly expressed in group 4 compared to groups 1 and 3 of our scRNA-seq UMAP analysis (but not group 2). Other contractility related genes overexpressed in group 4 include *TAGLN*, *MYL6*, *MYL9*, *MYL12A*, and *MYL12B* (Figure 4). Other overexpressed CT genes in group 4 compared with groups 1–3 included classic fibroblast markers such as *COL1A1*, *ELN*, *POSTN*, *THBS1*, *DCN*, *LUM*, and several drivers of fibrosis such as *TGFβ1*, *PDGFRB*, *TIMP-3*, *S100A4*, *GREM1* and *LOX* (Figure 4). The elevated level of *GREM1* in group 4 is striking when compared with the almost non-existent expression in groups 1–3.

**Figure 5:**
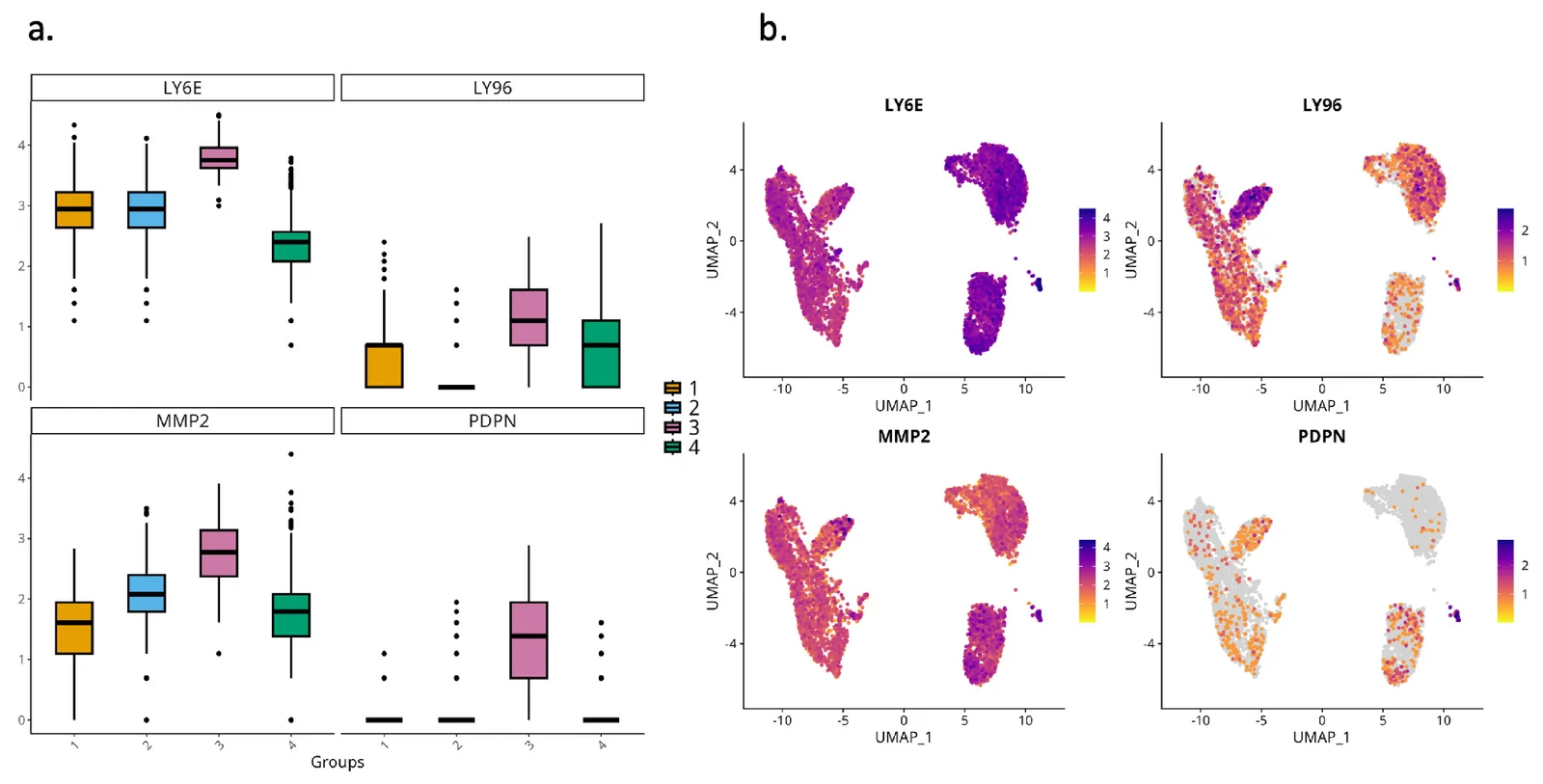
**Differential expression of ECM degrading genes across the 4 groups.** (**a**) Box plot of known ECM-degrading genes, including *PDPN*, *MMP2*, *LY6E*, and *LY96*. (**b**) Feature plot of corresponding ECM-degrading genes.

A recent report of scRNA-seq of CAFs in breast cancer has identified two key sub-groups, one of which is characterised by the overexpression of *S100A4*, the other by *PDPN* [32]. In our study, group 4 LC cells significantly overexpressed the calcium-binding protein *S100A4*, while group 3 LC cells (all from normal donors), overexpressed *PDPN*, a lymphatic glycoprotein marker that can mediate ECM degradation. Other ECM-degrading enzymes such as *MMP2* and *MMP14* also were overexpressed in group 3, as were members of the lymphocyte antigen family (*LY6E*, *LY96*, *LYPD6B*) and pro-inflammatory cytokines *IL6* and *IL33* (Figure 5a,b).

Overexpressed genes in group 1 included *LYPD1* (which is anti-angiogenic in heart fibroblasts and a putative tumour suppressor), *PCP4* (modulates calcium binding by calmodulin), *PCK1* (a key control point for gluconeogenesis), *SFRP1* (inhibits WNT signalling and is anti-proliferative) and *CDH6* (an adhesion cadherin).

The most overexpressed genes in group 2 included *IL13RA2* (or IL-13, which can drive fibrosis), *SERPINF1* (or *PEDF*, inhibits angiogenesis), *GAS1* (a growth arrest gene blocking entry to S-phase, a tumour suppressor), *PTGS2* (*COX2*, involved in the initiation of colorectal cancer), and *EFEMP1* (a secreted glycoprotein involved in cell adhesion and migration).

#### 2.1.5. GSEA Pathway Analysis in the Four Groups

**Figure 6:**
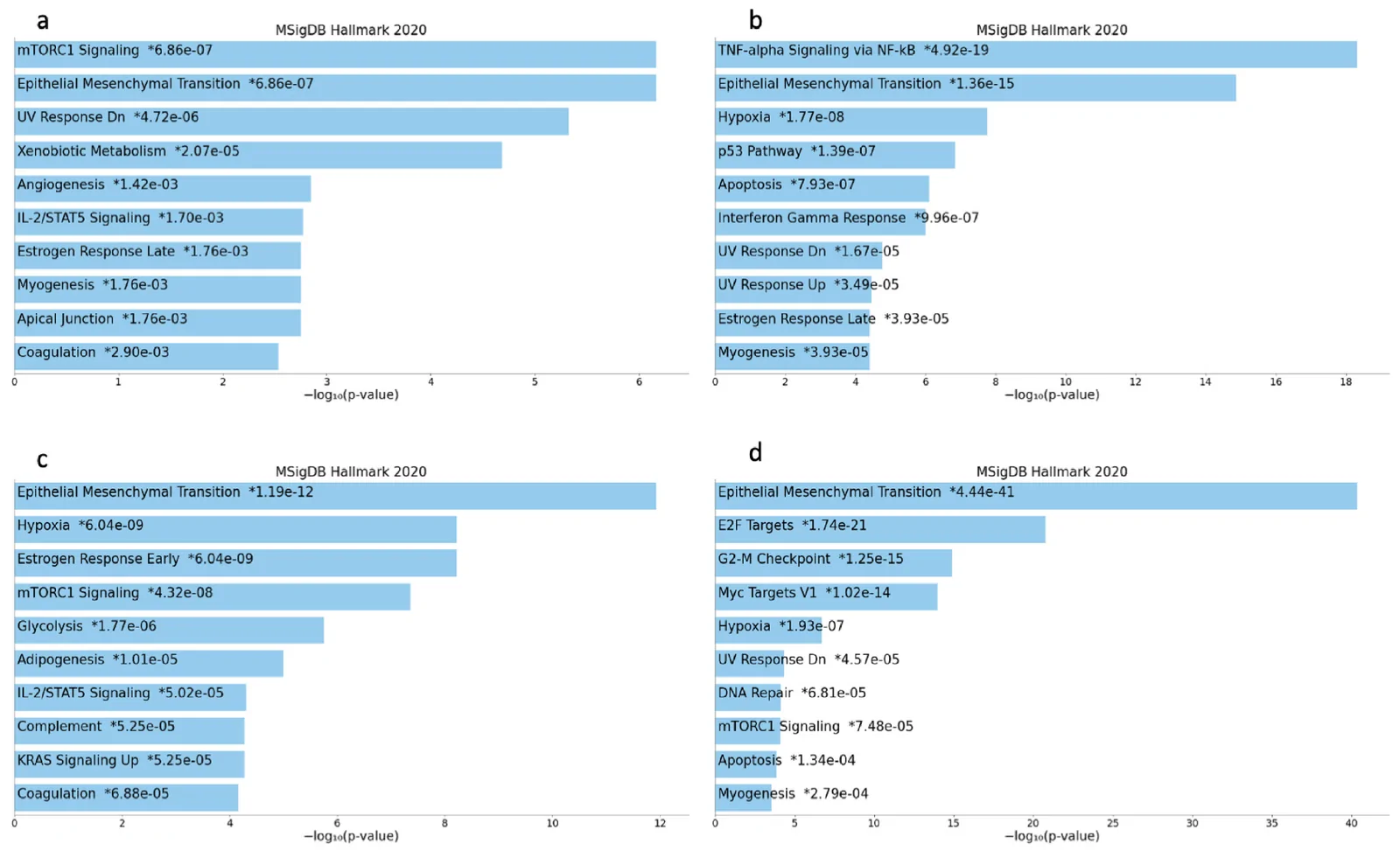
**Hallmark pathway analysis of preferentially expressed genes in each group.** (**a**) Hallmark pathway analysis of group 1 showing enrichment of mTORC1 signalling and the Hallmark EMT gene set. (**b**) Hallmark pathway analysis of group 2 showing enrichment of TNF-alpha signalling via NF-κB and the Hallmark EMT gene set. (**c**) Hallmark pathway analysis of group 3 showing enrichment of the Hallmark EMT gene set and Hypoxia pathway. (**d**) Hallmark pathway analysis of group 4 showing enrichment of the Hallmark EMT gene set and E2F targets. * *p* < 0.05.

To further characterise differences between the UMAP groups, we performed gene set enrichment analysis across the four groups individually using Enrichr and the Molecular Signatures Database (MSigDB) [30,31,32,33,34,35,36]. In group 1, composed entirely of normal donor cells, the mTORC1 signalling pathway appears highly upregulated (Figure 6a). In group 2, which was also composed entirely of normal cells, genes in the TNFα signalling via NF-kB pathway were preferentially expressed (Figure 6b). Group 3 showed a marked expression of the hypoxia signalling pathway (Figure 6c). Group 4, the predominantly glaucoma group, identified enrichment of E2F targets, a pathway for cell cycle regulation, as well as the G2M checkpoint and Myc Targets V1 pathways, two pathways which are highly relevant to cancer fibrosis (Figure 6d). Of note, the hallmark EMT gene set enriched across all four LC cell groups.

### 2.2. Normal Versus Glaucoma Data Analysis

#### 2.2.1. Gene Set Enrichment Analysis

**Figure 7:**
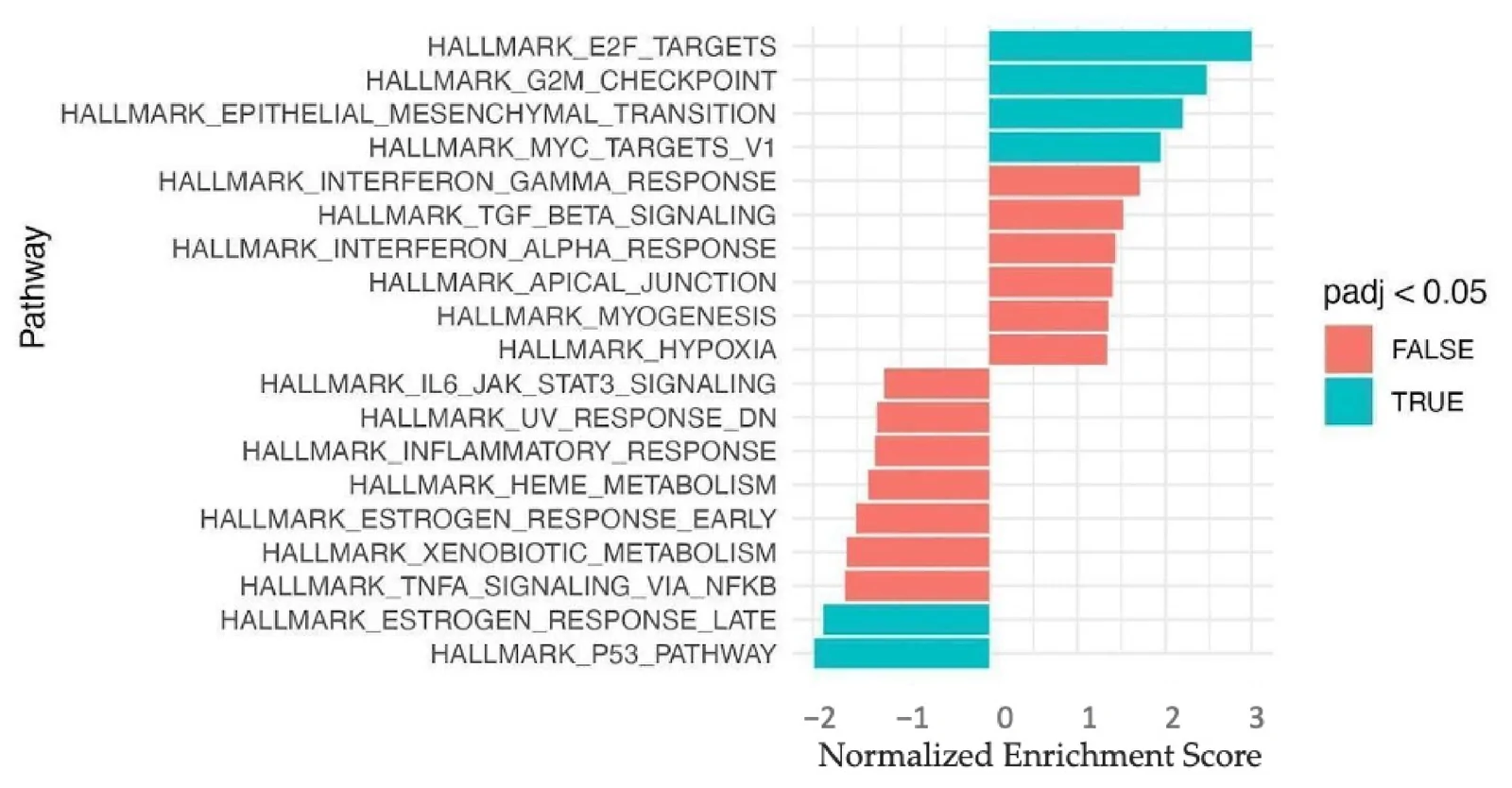
**Hallmark pathways upregulated and downregulated in glaucoma LC cells.** Ten most overexpressed and most negatively expressed hallmark pathways showing upregulation of E2F targets in glaucoma LC cells compared to normal LC cells, and showing downregulation of p53 pathway in glaucoma.

We compared gene expression between glaucoma and normal LC cells, showing that 823 genes were significantly overexpressed in glaucoma, and 1092 genes were significantly underexpressed in glaucoma (Supplementary Material S5). We then used the Hallmark gene set from the MSigDB and performed GSEA, which showed several hallmark pathways which were enriched in glaucoma (Figure 7). The most highly enriched pathway in glaucoma was the E2F target pathway, followed closely by the G2M checkpoint pathway, the Hallmark EMT gene set and MYC Targets V1. The E2F, G2M and MYC pathways are associated with cell-cycle regulation, while the Hallmark EMT gene set, which is well recognised in fibrosis and cancer, reflects an EMT-associated/profibrotic transcriptional programme characterised by increased expression of ECM-associated genes [37,38]. Other pathways significantly enriched in genes more highly expressed in glaucoma include the interferon gamma response (also known as the Jak-Stat pathway), and the TGFβ signalling pathways. The most downregulated pathways were the p53 pathway and oestrogen response late pathway. The significantly reduced p53 pathway could lead to reduced fibroblast apoptosis—a prominent feature of multiple forms of systemic fibrosis [39].

To assess the robustness of the original differential-expression findings, additional donor-level pseudobulk analysis was performed using DESeq2. This identified 767 significantly differentially expressed genes (adjusted *p* < 0.05). The complete donor-level pseudobulk differential-expression results, including log2 fold changes, standard errors, Wald statistics and adjusted *p*-values, are provided in Supplementary Material S6. Comparison with the original MAST results demonstrated substantial concordance (Spearman’s r = 0.78), with 94.4% of genes changing in the same direction. Further filtering identified 688 significantly differentially expressed genes exhibiting concordant changes of at least twofold in both glaucoma donors G2 and G3 relative to normal donors (Supplementary Material S7).

Pathway enrichment analysis demonstrated preservation of the principal disease-associated transcriptional programmes, including extracellular matrix organisation, EMT-associated/profibrotic signalling and TGF-β-associated pathways. A further sensitivity analysis was performed by excluding glaucoma donor G3, which had been processed in a separate sequencing run, and repeating MAST differential-expression analysis. Although individual differentially expressed genes varied following exclusion of this donor, enrichment of the principal extracellular matrix, fibroblast, TGF-β, hypoxia and EMT-associated pathways was retained. These findings support the principal pathway-level interpretation while highlighting sensitivity of individual gene-level results to donor inclusion. Supporting results from the G3-exclusion analysis are provided in Supplementary Material S8. Removal of putative within-donor doublets had minimal influence on differential-expression estimates (Spearman’s r = 0.999), indicating that the original findings were not materially affected by predicted within-donor doublets. Donor-specific doublet counts and supporting sensitivity-analysis results are also provided in Supplementary Material S9.

#### 2.2.2. Orthogonal Testing of Differential Gene Expression

**Figure 8:**
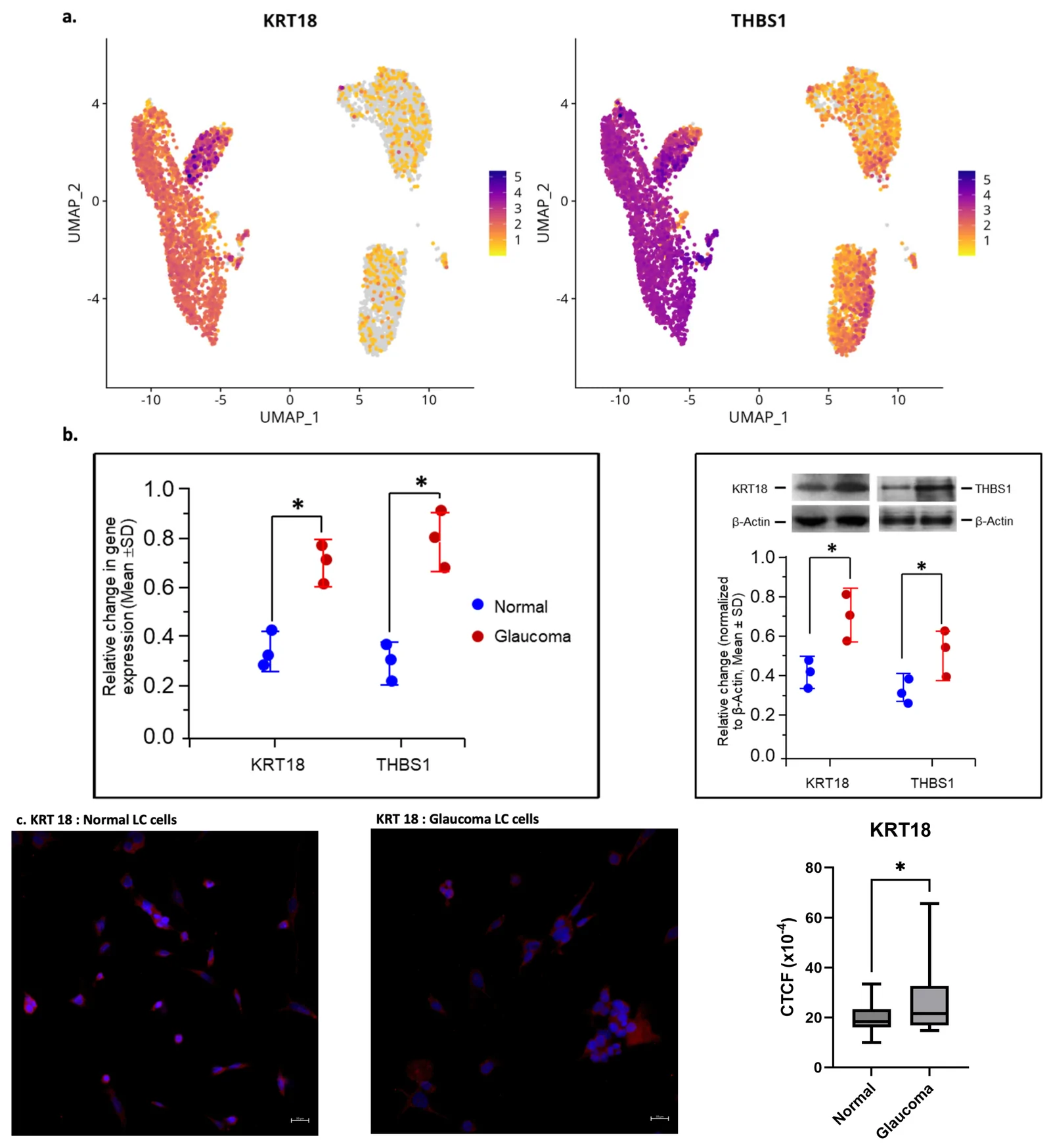

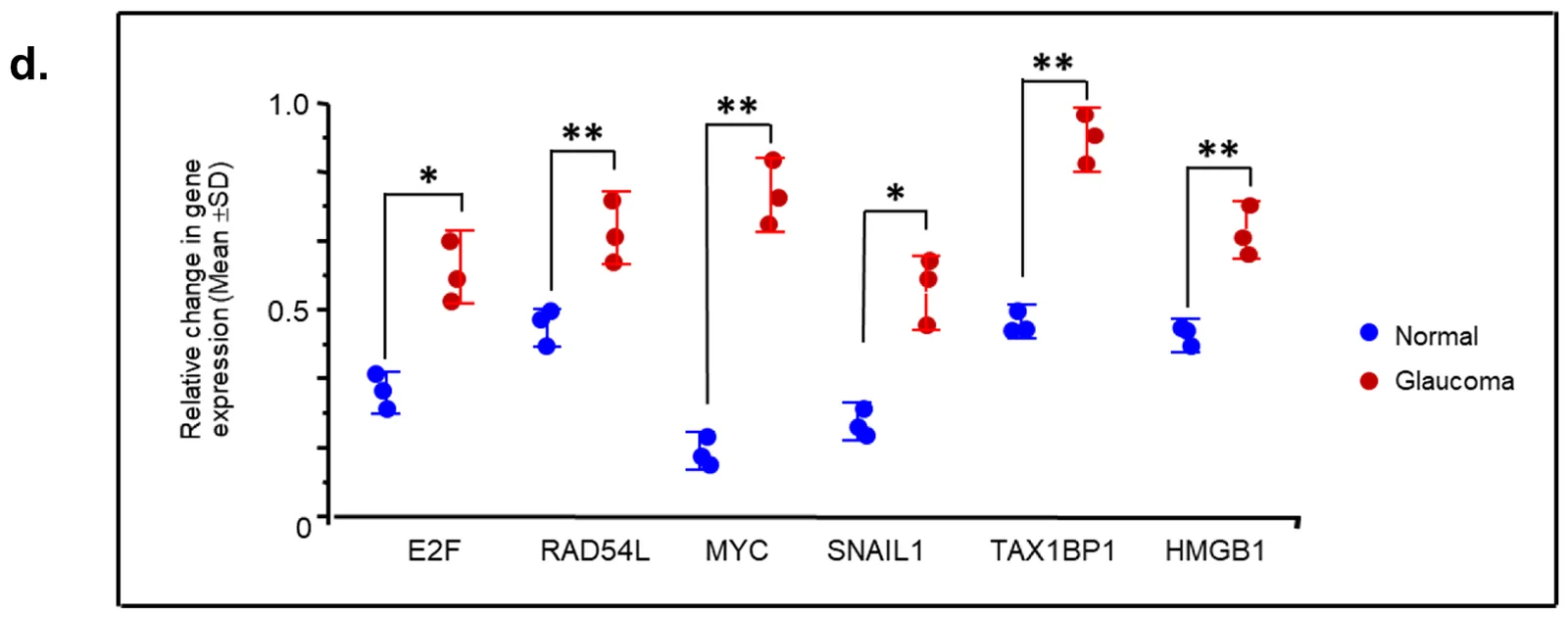
**Differential expression and levels of selected genes and proteins in glaucoma (using RT-qPCR, WB and IF).** (**a**) UMAP plots of gene expression of *KRT18* and *THBS1* showing an increase in group 4. (**b**) RT-qPCR and WB of *KRT18* and *THBS1* gene expression and protein levels in NLC and GLC. (**c**) IF of KRT18 LC cells showing increased staining in cytosol of glaucoma LC cells, with quantification of immunofluorescence adjacent. (**d**) Expression of *E2F1*, *RAD54L*, *MYC*, *SNAI1*, *TAX1BP1* and *HMGB1* in normal and glaucoma LC cells using RT-qPCR. * *p* < 0.05, ** *p* < 0.02.

We used RT-qPCR, WB and IF to test the scRNA seq data using independent donors to the donors used in the scRNA-seq. First, gene expression and protein levels of *KRT18* and *THBS1* were assessed in LC donor cells, as well as by IF for *KRT18*, and all were shown to be elevated in glaucoma compared to normal. (Figure 8a–c). We also performed RT-qPCR to analyse the levels of representative genes in LC cells from the GSEA pathways, and showed that these were increased in glaucoma (*E2F1* [E2F pathway], *RAD54L* [G2M pathway], *MYC* and *SNAI1* [Hallmark EMT gene set] (Figure 8d). Additional candidate genes were evaluated during the course of the study, such as *VTN* and *SBSPON*, but these did not show consistent changes between normal and glaucomatous lamina cribrosa cells. In addition, treatment of glaucomatous cells with tPA was explored; however, this did not result in a reduction in the expression of the genes of interest.

### 2.3. Transcription Factor Analysis

We used a network analysis to ascertain which TFs were enriched in glaucoma cells. We inferred a gene regulatory network using fibroblast datasets (>2000 samples) downloaded from the ARCH4 database and the GENIE3 algorithm [40,41]. Next, we analysed glaucoma markers from our scRNA-seq results using this network and the Viper algorithm, which identified master regulons (Table 1). Amongst the significantly overexpressed TFs involved in the cell cycle were HMGB1 and 2, which have been hypothesised as novel targets for cancer treatment, as well as CENPA, which, in the presence of decreased p53, acts as a promoter of EMT and tumour cell invasion [42,43]. Significant downregulation of TCF25, a key element of the ribosome-associated quality control complex, was also seen [44]. The full gene list for each regulon is shown in Supplementary Material S10.

### 2.4. i-cisTarget and the Integrated Stress Response

i-cisTarget is an integrative genomics method for the prediction of regulatory features and cis regulatory modules [45,46]. Cis regulatory modules (CRMs) are regions of non-coding DNA that have transcriptional regulatory activity over neighbouring genes. CRMs allow binding of active TFs and co-factors to mediate regulation of gene expression. By contrasting the data from our glaucoma LC cells and normal LC cells we found 315 enriched TF regions with normalised enrichment score (NES) > 3 (Supplementary Material S11). The most highly represented CRM/TFs included serum response factor (SRF; 25 of the top 30 enriched regions) and the MEF2 (A-D) family, both members of the MADS box superfamily of TFs, which bind to the serum response element (SRE) in the promoter region of target genes involved in cell proliferation [47]. We tested SRF levels and found an increase in glaucoma LC cells using PCR. In addition, the AP-1 family of TFs were highly enriched in glaucoma, including *FOSL2*, *JUN*, *AP-1*.

**Figure 9:**
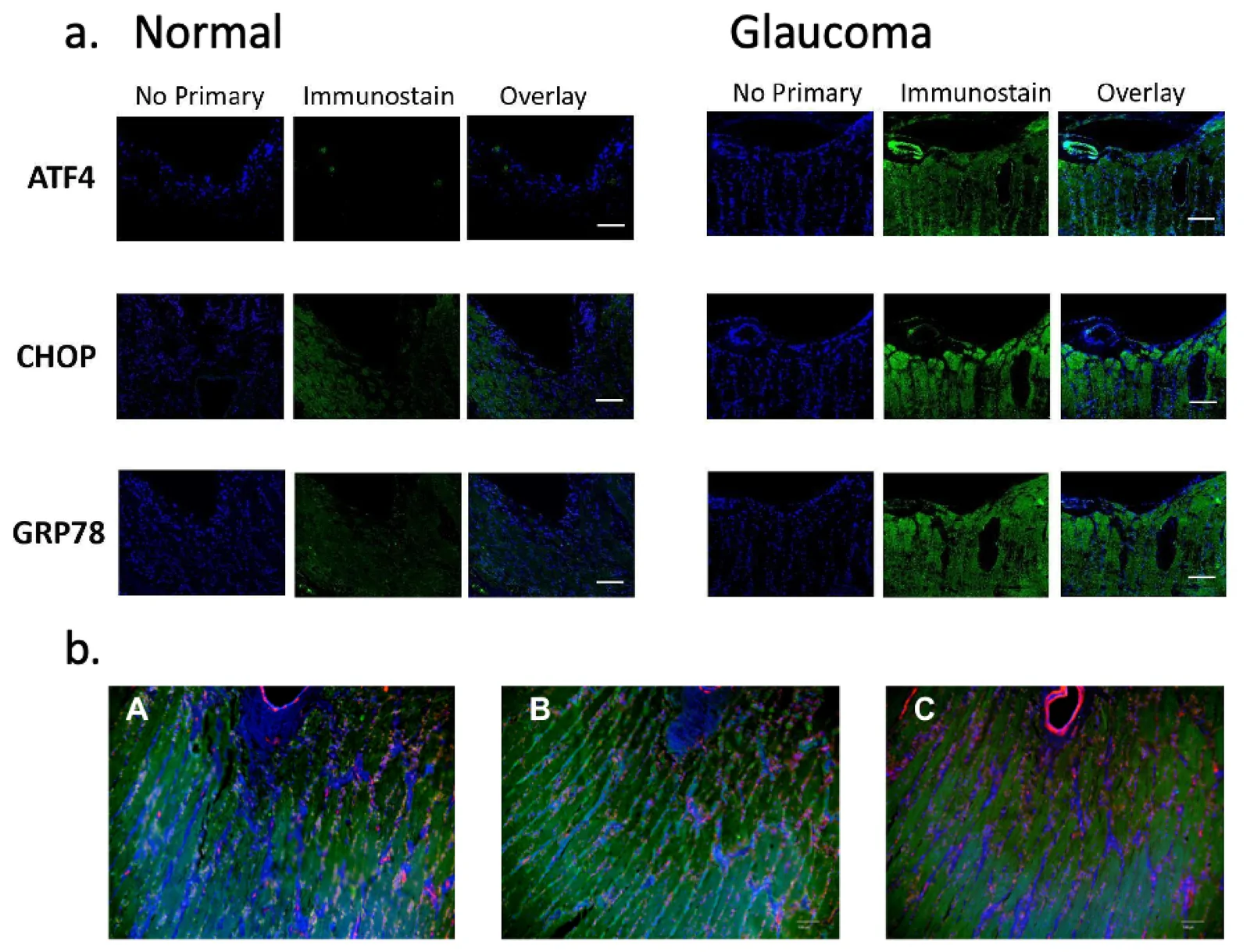
**Immunohistochemistry—staining for ATF4, CHOP, GRP78.** (**a**) IHC of normal and glaucoma ONH tissues (*n* = 3–6) immunostained for control, ATF4, CHOP, and GRP78. (**b**) Immunohistochemical co-staining of ONH tissue from a glaucoma donor for αSMA (red) as well as (green): (**A**) GRP78, (**B**) CHOP, and (**C**) ATF4. Sections were counter-stained with DAPI (blue).

**Figure 10:**
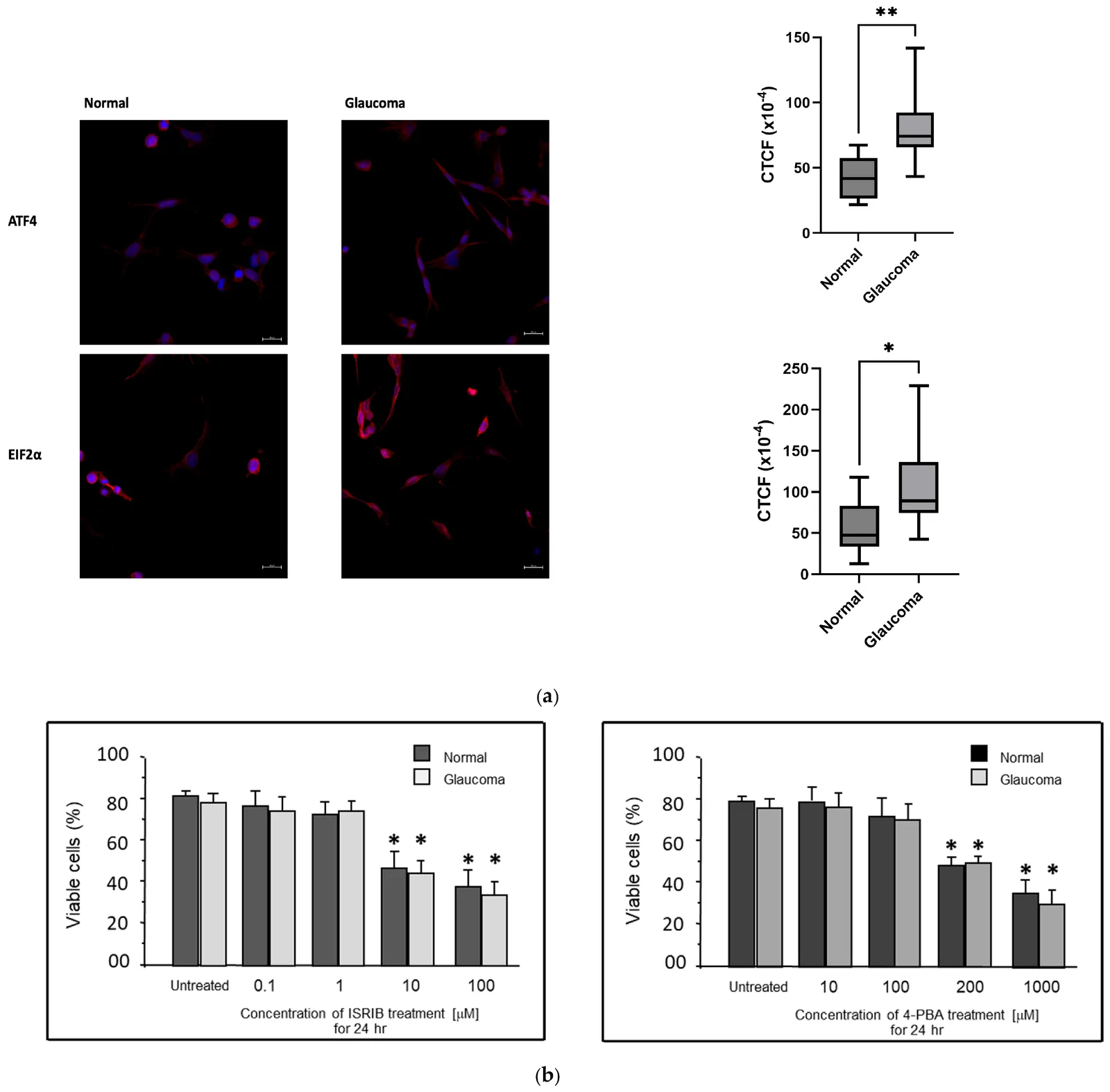

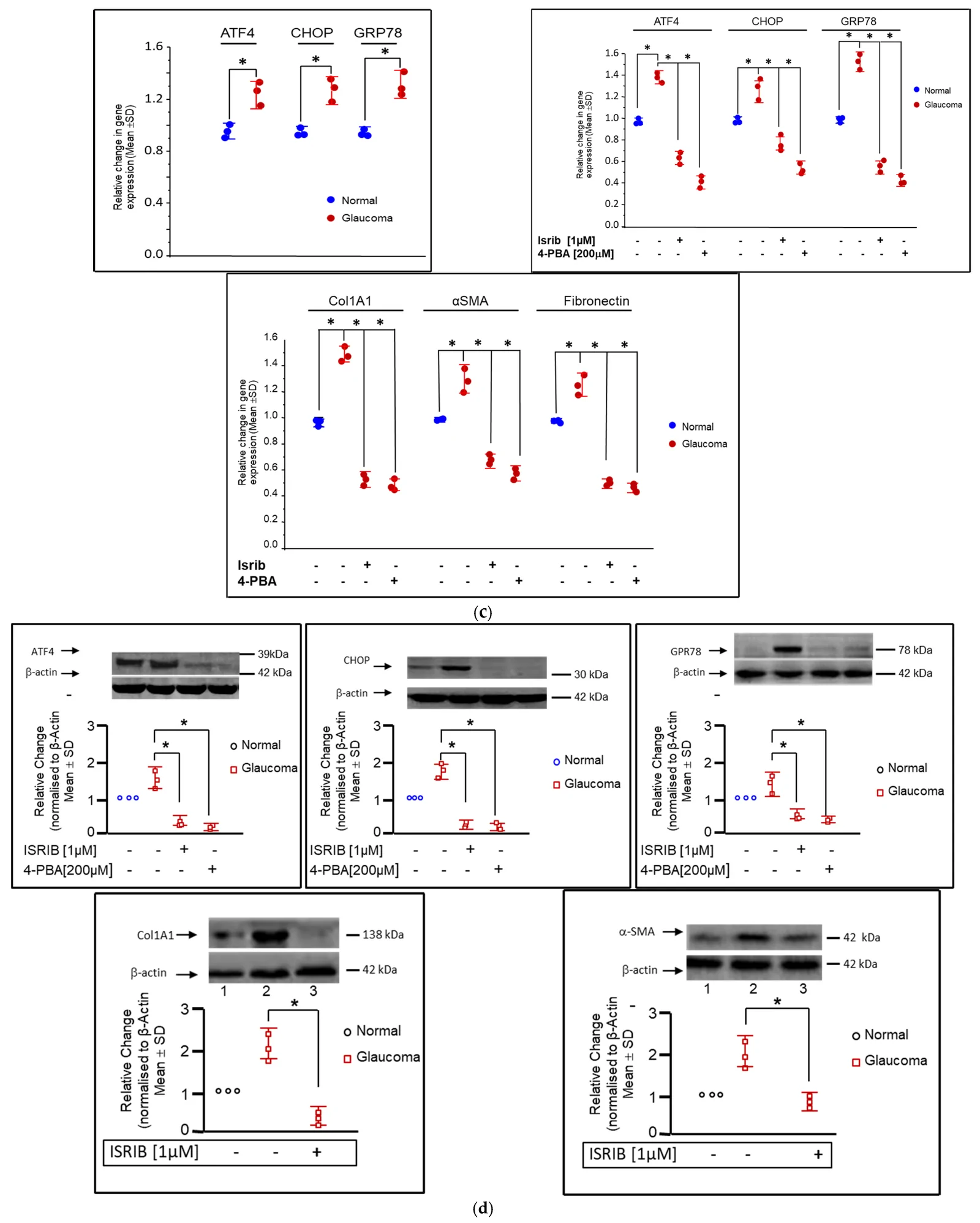
**Expression and levels of ISR and ECM specific genes, protein.** (**a**) IF of ATF4 and pEIF2α (phosphorylated EIF2α) in normal and glaucoma cells with quantification of immunofluorescence levels adjacent (**b**) MTT-derived cell viability following treatment with increasing concentrations of ISRIB and 4-PBA. (**c**) Gene expression of ISR/UPR related genes (*ATF4*, *CHOP*, *GRP78*) from normal, glaucoma, ISRIB treated and 4-PBA treated LC cells showing increased expression in glaucoma and decreased expression post both treatments. (**d**) Western immunoblot of ISR related proteins (ATF4, CHOP and GRP78) and ECM related proteins (Col1A1, αSMA) in normal, glaucoma and glaucoma treated with ISRIB and 4-PBA showing increased abundance in glaucoma and decreased abundance post treatment. * *p* < 0.05. ** *p* < 0.02.

A homolog of *ATF4*, *GCN4*, was found to be overexpressed using i-cisTarget, as well as *ATF3*, *CREB3L1* and *ATF6b*, all key components of the endoplasmic reticulum/unfolded protein response/integrated stress response (ER/UPR/ISR) pathways [48,49]. The UPR has evolved in cells to eliminate misfolded/unfolded proteins via autophagy and ER-associated protein degradation (ERAD) [50]. The UPR signals through three potential routes; *ERN1(IRE1)*, *PERK* and *ATF6*. *GRP78* plays a key role in the control of the UPR, macroautophagy, cell survival and proliferation [51]. Harding et al. demonstrated that chronic ER (unfolded protein) and oxidative stress plus amino acid starvation triggers the phosphorylation of *eIF2α*, which inhibits general protein translation/synthesis, while activating the TF *ATF4*, a central node in the ISR [52,53]. We used RT-qPCR, IF and IHC to test several key ISR/UPR genes (*ATF4*, *CHOP*, *GRP78*) and showed that these were increased in glaucoma LC cells as well as human glaucoma ONH tissues (Figure 9 and Figure 10). Increased expression of these three stress markers in the glaucomatous ONH was associated with co-expression of LC cells expressing αSMA (Figure 9b). Furthermore, a review of the group 4 data found elevated levels of several genes from the eukaryote translation initiation factor (eIF) family, including *eIF2s2 (eIF2B)*, *eIF4A1*, *eIF5A*, and *eIF1AX*. We showed an increase in p-EIF2α by IF analysis (Figure 10a). *eIF2B* is the target for the ISR inhibitor ISRIB [54].

#### 2.4.1. Pharmacological Modulation of ISR/ER-Stress-Associated Signalling

Zode’s group found elevated *GRP78* in glaucoma TM cells, and later demonstrated a pathological role for chronic ER stress-induced ATF4-CHOP signalling leading to trabecular meshwork cell dysfunction, elevated IOP and retinal neurodegeneration in a mouse model of glaucoma [55,56]. ISRIB has also recently been shown to effectively mitigate liver fibrosis [57]. Given the increased ISR/UPR markers observed in glaucomatous LC fibroblasts, we investigated the effects of pharmacological modulation using the ISR inhibitor ISRIB and the ER stress inhibitor 4-PBA. Prior to assessing the effects of ISRIB and 4-PBA on ISR/UPR and ECM-related markers, the potential cytotoxicity of the treatments was evaluated using an MTT cell-viability assay. The selected experimental concentrations of ISRIB (1 µM) and 4-PBA (200 µM) were within the concentration ranges in which cell viability was maintained. For 4-PBA, concentrations of 10–200 µM maintained MTT-derived viability above 91% of control, whereas viability decreased at higher concentrations. Similar dose–response experiments were performed for ISRIB (Figure 10b). As MTT is a metabolism-dependent tetrazolium assay, maintenance of MTT signal supports the absence of a marked reduction in metabolic viability at the selected concentrations but does not exclude all forms of nonspecific cellular toxicity.

Treatment with either agent reduced the level of ISR/UPR-related proteins (Figure 10c,d). At baseline, ATF4, CHOP, and GRP78 protein levels were elevated in glaucomatous LC cells compared to controls (ATF4: 1.22 ± 0.192 vs. 1; CHOP: 1.62 ± 0.243; GRP78: 1.29 ± 0.166; *p* < 0.05, *n* = 3). Pre-treatment with ISRIB or 4-PBA decreased levels of all three proteins (ATF4: 0.186 ± 0.083 and 0.12 ± 0.028; CHOP: 0.182 ± 0.039 and 0.12 ± 0.037; GRP78: 0.59 ± 0.057 and 0.38 ± 0.052, respectively).

We next assessed ECM-related gene expression and protein levels (COL1A1, ACTA2, FN1) (Figure 10c,d). Glaucomatous LC cells showed increased Col1A1 and α-SMA protein levels compared to normal controls (Col1A1: 2.58 ± 0.42 vs. 1; α-SMA: 2.23 ± 0.38; *p* < 0.05, *n* = 3). Treatment with ISRIB reduced the level of these proteins present (Col1A1: 0.34 ± 0.083; α-SMA: 0.75 ± 0.076), with similar trends observed following 4-PBA treatment. Data are presented as mean ± SD. It is important to note that treatment with both agents decreased the expression of all genes below normal levels, and in the future further dose response experiments would need to be conducted.

#### 2.4.2. Effect of ISRIB on Cellular Metabolic Activity in Glaucoma LC Cells

**Figure 11:**
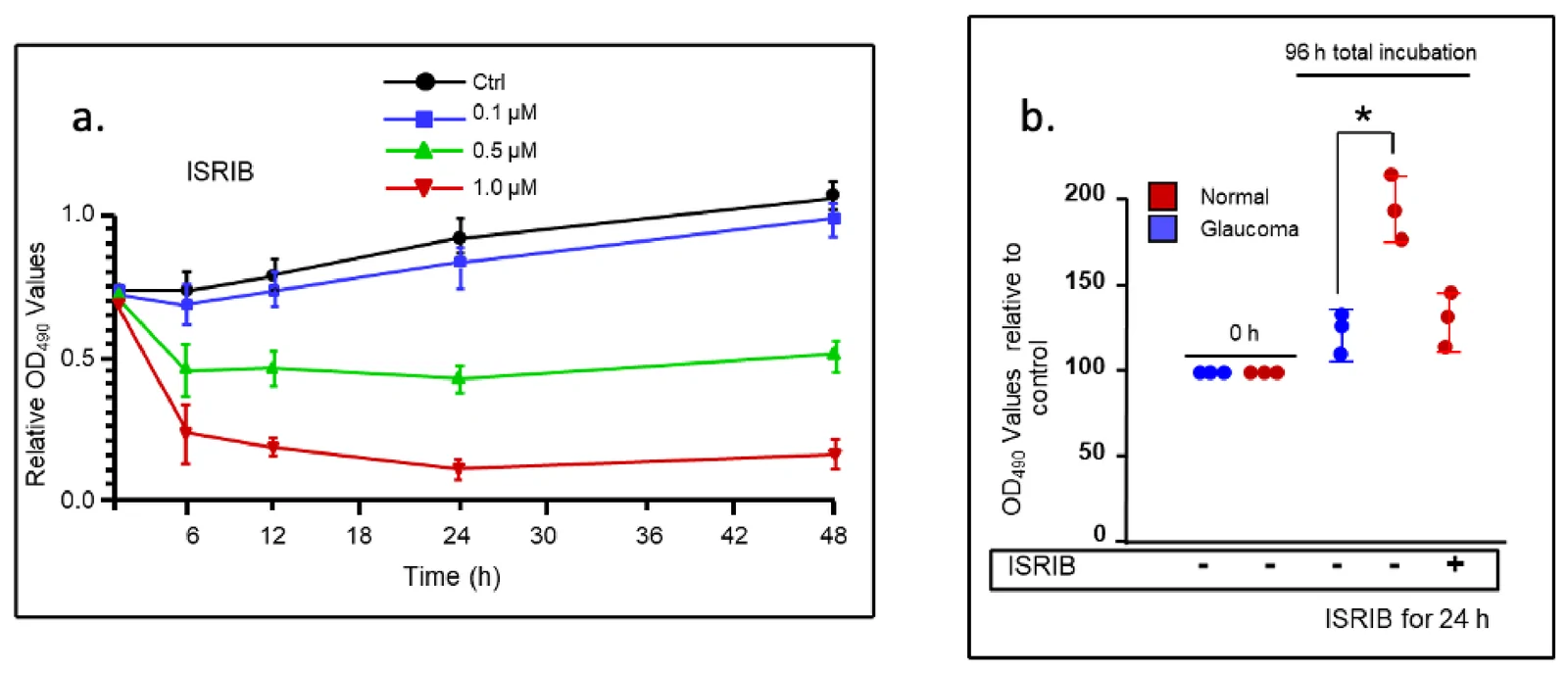
**Effect of ISRIB on cellular metabolic activity in glaucoma.** (**a**) Effect of ISRIB on cellular metabolic activity in glaucomatous lamina cribrosa (LC) cells assessed using the colorimetric MTS assay. MTS-derived cellular metabolic activity was assessed at 0, 6, 12, 24 and 48 h following treatment with ISRIB at 0 μM (control), 0.1 μM, 0.5 μM and 1.0 μM. ISRIB treatment resulted in a dose- and time-dependent reduction in MTS-derived cellular metabolic activity, with the most marked effect observed with 1.0 μM ISRIB at 24 h. Therefore, 1 μM ISRIB was used for subsequent experiments. Untreated cells were used as controls and the mean OD of the control was set to 100%. Data represent mean ± SEM from three independent donors (*n* = 3), with each donor analysed in triplicate and technical replicates averaged to generate a single donor-level value prior to statistical analysis. (**b**) MTS-derived cellular metabolic activity was significantly increased in glaucomatous LC cells (GLC) compared with normal LC cells (NLC) at 96 h (193.4 ± 21.4% vs. 124.2 ± 13.7%; *p* < 0.05, *n* = 3). Treatment of GLC cells with 1 μM ISRIB for 24 h reduced relative MTS-derived cellular metabolic activity to 129.2 ± 16.9% (*p* < 0.05, *n* = 3). * *p* < 0.05 versus the corresponding control.

To investigate the impact of ISRIB treatment on cellular metabolic activity in normal and glaucoma LC cells, we first applied different concentrations of ISRIB (0.1 μM, 0.5 μM, and 1.0 μM) to the cells for varying durations (6 h, 12 h, 24 h, and 48 h). While no significant effect on MTS-derived metabolic activity was observed with 0.1 μM ISRIB compared with the untreated control group, both 0.5 μM and 1.0 μM ISRIB treatment resulted in reduced MTS-derived metabolic activity, with the most marked effect observed at a concentration of 1.0 μM after 24 h. The MTS assay results (Figure 11a) demonstrated a dose- and time-dependent reduction in cellular metabolic activity following ISRIB treatment. MTS-derived metabolic activity in NLC and GLC cells was similar at 72 h, but was significantly increased in GLC cells compared with NLC cells at 96 h (193.4 ± 21.4% vs. 124.2 ± 13.7%; *p* < 0.05, *n* = 3). ISRIB treatment of glaucoma cells reduced the relative MTS signal to 129.2 ± 16.9% (*p* < 0.05, *n* = 3) (Figure 11b).

## 3. Discussion

### 3.1. Analysis of scRNA-Seq

The unsupervised cluster analysis of the scRNA-seq of all LC cells found four distinct groups. Groups 1 and 2 were an admixture of the two normal donors, each characterised by distinct GSEA signalling pathways, predominantly mTORC1 for group 1, and TNF alpha signalling via NFkB for group 2. Group 3, the furthest group from group 4, overexpressed several ECM degrading enzymes such as *PDPN*, *MMP2* and *MMP14*. Group 4 cells were from the three glaucoma donors, with overexpression of multiple classic ECM (*COL1A1*, *POSTN*), contractile (*TAGLN*, *MYL9*), and pro-fibrotic (*TGFB1*, *LOX*) genes. Expression of the profibrotic gene *GREM1* also was elevated in group 4, in agreement with a previous report of increased *GREM1* in glaucoma ONH cells [58]. Notably, all four groups expressed multiple markers of activated contractile fibroblasts (*ACTA2*, *COL1A1*, *LOX*), which was supported by enrichment of the Hallmark EMT gene set, interpreted here as an EMT-associated/profibrotic transcriptional programme rather than classical epithelial-to-mesenchymal transition. Of note, cells from each of the normal donors were seen across all four groups, suggesting that some of the normal cells express myofibroblast markers. This is not entirely surprising given that the LC is constantly exposed to biomechanical stress and strain throughout life [6], even in ‘normal’ eyes due to diurnal variation in IOP, the pulsation of IOP at the LC with each cardiac pulse, and the increase in LC stiffness with age [59]. The relative lack of ECM-degrading enzymes may be a key factor leading to LC fibrosis and stiffness in the glaucoma cells.

Analysing the GSEA pathways across the four groups highlighted significantly different expression pathways. The characterisation of both normal donors found in group 1 (mTORC1) and group 2 (TNF alpha signalling via NFkB) signalling pathways might suggest that these two signalling pathways are somehow “protective” against the conversion of fibroblasts to activated contractile myofibroblasts. mTORC1 activation has been shown to regulate TGFβ-induced myofibroblast metabolism and collagen synthesis [60]. TNFα accelerates the resolution of established pulmonary fibrosis in mice [61].

Bioinformatic analysis of public databases has shown an over-representation of pro-fibrotic pathways in glaucoma TM, LC and Tenon’s fibroblast cells [62,63,64]. Also, a recent report that combines population-based GWAS with scRNA-seq in glaucoma-relevant ocular tissues demonstrates an overexpression of ECM/CT genes in glaucoma, and a focus on fibroblasts in the anterior and posterior segments of the eye including the ONH and peripapillary sclera [11]. The current study confirms this pro-fibrotic phenotype by the overexpression of the EMT pathways in glaucoma compared with normal patients.

Cell type analysis demonstrated that the LC cell studied in this investigation was mesenchymal/fibroblast in origin. This confirms the initial characterisation studies by Hernandez and Clark of cells that express *ACTA2*, *FN1* and *COL1A1*, as well as the absence of markers for the other cell types (astrocytes, microglia, oligodendrocytes) found in the human ONH [15,18]. A recent scRNA-seq analysis of the human ONH identified individual clusters of astrocytes, vascular endothelial cells, and a single cluster of fibroblasts [26]. The ONH fibroblast cluster may represent these LC fibroblasts. The functional heterogeneity and molecular markers of fibroblasts and myofibroblasts found in fibrosis and cancer using scRNA-seq analysis has been comprehensively covered in recent reviews [65,66]. Two recent scRNA seq studies of human TM cells have identified significant clusters of fibroblasts and myofibroblasts among a number of cell types present [25,67].

We acknowledge that all LC cells were analysed following ex vivo culture, which may influence transcriptional states and contribute to the observed heterogeneity. Cells were used at early passage (≤passage 8), under conditions in which LC fibroblasts have previously been shown to retain stable morphology and disease-relevant gene expression [68,69,70,71]. Nevertheless, effects of substrate, serum exposure, and loss of the native 3D microenvironment cannot be excluded. Prior studies of LC fibroblasts and other primary fibroblast systems similarly support relative stability of early-passage cultures, with greater transcriptional drift at later passages [72,73,74]. Consistent with this, we observed preserved LC fibroblast markers and no evidence of non-fibroblast lineages. We therefore interpret the groups as reflecting a combination of donor-associated states and culture-modulated variation. Definitive in vivo validation will require spatial transcriptomic or RNAscope approaches in intact optic nerve head tissue, which remain technically limited in this region.

### 3.2. Gene Set Enrichment Analysis: Cell Cycle Proliferation and Apoptosis Pathways

The mammalian cell cycle is divided into four phases: mitosis, DNA synthesis, G1 and G2. The G1/S phase for DNA synthesis is activated by the E2F TFs, while the mitosis phase is activated by the G2M TFs [75]. Having previously reported an increased rate of proliferation in glaucoma LC cells [76], we found the overexpressed GSEA pathways (E2F, G2M, c-MYC) in glaucoma to be of particular relevance. The E2F pathway includes TFs that play a key role in cell cycle progression and proliferation, and cancers with high E2F signatures have a poorer prognosis [77]. The G2M checkpoint pathway was highly enriched, a pathway which normally prevents cells from entering mitosis when DNA is damaged. This pathway was highlighted by enrichment of genes including *STMN1*, an oncoprotein crucial for the regulation of the cell cytoskeleton, as well as *MCM3* and *MCM6*, highly conserved mini chromosome maintenance proteins [78]. A recent study reported that human fibrotic pulmonary fibroblasts expressing POSTN and GREM1 also show enrichment of proliferative signatures, including elevated G2M pathway activity [79]. These observations suggest that proliferative transcriptional programs may be a feature of activated fibroblast populations in fibrotic disease contexts.

The MYC Targets V1 pathway was also a significantly enriched pathway in the glaucoma group; this is a well known pathway in cancer biology which activates many pro-proliferative genes [80]. MYC Targets V1 was highlighted by the enrichment of *NME1*, *SSBP1*, and *EIF2S2*. MYC is an oncogene TF involved in proliferation, growth, metabolism, DNA replication and cell cycle progression [80]. MYC can also downregulate cell cycle inhibitors or “brakes”, such as the p27 (kip1) [81], which we have previously identified in glaucoma LC cells, thereby contributing to increased proliferation [82].

The p53 pathway had the most negative GSEA pathway score and lends support to our recent observation of reduced p53 protein abundance and increased abundance of the E3 ubiquitin-protein ligase MDM2 in glaucoma LC cells [83]. The failure of myofibroblast apoptosis and therapy targeting the p53 pathway is under investigation in all forms of fibrosis [39].

### 3.3. Transcription Factor Analysis

Using the GENIE3 and VIPER algorithms, our analysis identified several over-represented ‘regulons’, including HMGB1 and 2, and CENPA, which are known to be involved in cancer cell proliferation [84,85]. The most frequently identified TF in our i-cisTarget analysis was SRF, which is known to regulate gene activation at specific stages of cell growth (the G0–G1 transition and G2M) and is involved in actin cytoskeleton expression [86]. The MRTF/SRF pathway plays a key role in activating myofibroblasts, and in an ex vivo study, inhibitors of this pathway were shown to decrease conjunctival fibrosis post glaucoma filtration surgery [87]. *MEF2A*, *MEF2B*, *MEF2C* and *MEF2D*, members of the MADs box superfamily, were also enriched. The AP-1 family of TFs also frequently appeared in our analysis. This family has been shown to be activated in response to oxidative and ER stress, and further linked to proliferation and transformation of tumour cells [88]. AP-1 has previously been linked with glaucoma pathogenesis on integrated microarray analysis [89]. The many connections between genes of interest identified through a variety of techniques is illustrated graphically using the String database (Supplementary Material S12).

### 3.4. Endoplasmic Reticulum Stress and the Integrated Stress Response

The i-cisTarget motif target analysis identified *GCN4* (*ATF4* homologue) to be overexpressed in glaucoma LC cells, as well as several other ER stress and ISR-related genes including *CREB3L1*, *ATF3* and *ATF6b* [90]. We confirmed that several components of the ATF4 –ISR pathway (*ATF4*, *CHOP*, *GRP78*, *p-eIF2α*) were overexpressed in glaucoma LC cells. Inhibition of the ER stress and ISR pathways using 4-PBA and ISRIB, respectively, reduced ER/ISR markers, significantly reduced several key fibrotic ECM genes and proteins, and reduced the increased cellular metabolic activity in glaucoma cells. Chronic cell stress factors, either extrinsic (hypoxia, low amino acid levels, low glucose) or intrinsic (ER stress secondary to unfolded proteins), leads to an inhibition of general protein translation and an increase in ATF4/CHOP—the ISR pathway [52,53]. *ATF4* can also regulate autophagy and the expression of genes involved in amino acid synthesis and transport. In CAFs, *ATF4* controls the expression of *COL1A1* (the predominant collagen in ECM), and levels of glycine and proline—the major amino acids in collagen [91]. In addition, *ATF4* has been shown to couple MYC-dependent translational activity to bioenergetic demands during tumour progression [92].

ER stress associated with protein misfolding in myocilin-related early onset glaucoma has been recognised for many years [93]. Zode more recently identified ER stress in TM cells in adult onset POAG and noted that genetic depletion or pharmacological inhibition of the ATF4-CHOP-GADD4 pathway prevents TM cell death and rescues mouse models of glaucoma [27,28,55,56]. Fang et al. demonstrated that RGC-specific deletion of CHOP or ATF4 synergistically promotes RGC and ON survival in optic nerve crush and silicone oil induced ocular hypertension mouse models [94]. Shiga et al. showed that ER stress is a key feature of glaucomatous neurodegeneration, and that SERCA2 gene supplementation, which reduces ER stress, restores visual behaviours in mice, and promotes neurorecovery [95].

ISRIB is a potent inhibitor of the ISR, making cells resistant to the effects of eIF2α phosphorylation, and is being studied in neurodegeneration, demonstrating an improvement in long term memory of treated rodents and a reduction in concussion related head trauma in mice [96,97]. ISRIB decreases IOP in mouse models of glaucoma, and decreases ISR-related gene expression (*ATF4*, *CHOP* and *GADD34*) in TM cells when applied topically [56], but the effects on LC cells have not been previously studied. 4-PBA is a small chemical chaperone and histone deacetylase inhibitor first discovered for the treatment of urea cycle disorders, and now being studied in the treatment of cystic fibrosis and several cancers [98]. 4-PBA has previously been shown to rescue mouse models of glaucoma through reduction in ocular ER stress, and to decrease ER stress-related gene expression in RGCs [27,28,99].

Limitations of the study include a lack of clinical information (disease severity, duration, and treatment) for the patient donors, and a scarcity of available donor material. Translation of our findings to mouse or rat models of glaucoma is problematic due to the lack of a true LC at the ONH. Rodents instead have a glial lamina composed of ONH astrocytes, and rodent LC cells have yet to be found (if they exist). Johnson et al. have shown increased cell proliferation and increased expression of ECM genes at the optic nerve head in sustained IOP elevation in a rat model [100]. Reactivated astrocytes also play a significant role in optic nerve head fibrosis in rodent models of glaucoma [101]. Detailed analyses addressing ER stress specificity, including controls related to HDAC activity or histone acetylation, were not included in the present study, but represent important directions for future investigation.

An important limitation of the scRNA-seq analysis is the small number of biological donors (two normal and three glaucoma). Differential-expression analysis using MAST was performed at the individual-cell level without donor identity included as a covariate. However, additional donor-level pseudobulk analysis demonstrated substantial concordance with the original results, and sensitivity analyses supported preservation of the principal biological pathways. Nevertheless, the limited number of donors restricts statistical power and generalisability, and individual gene-level results remain sensitive to donor inclusion. Furthermore, because the third glaucoma donor was processed in a separate sequencing run, sequencing-run effects cannot be reliably distinguished from donor-specific biological variation. The scRNA-seq findings should therefore be interpreted cautiously, with candidate genes and pathways prioritised for validation in larger independent donor cohorts.

A further limitation is that scRNA-seq was performed on cultured, expanded LC cells rather than freshly isolated LC tissue. The identified groups therefore represent transcriptional states within cultured LC cells and should not automatically be interpreted as native in vivo LC fibroblast subpopulations. Culture conditions, serum exposure, substrate stiffness, loss of the native three-dimensional ECM environment and absence of physiological mechanical loading may influence LC-cell transcriptional state, as demonstrated by our lab previously.

The MTS assay measures tetrazolium reduction and therefore reflects cellular metabolic activity/viability rather than directly measuring proliferation. It cannot distinguish reduced proliferation from altered cellular metabolism or cell death. Proliferation-specific assays such as EdU/BrdU incorporation or Ki-67 assessment would be required to establish a direct effect on proliferation.

## 4. Methods

### 4.1. Cell Culture

Primary cultures of human lamina cribrosa (LC) cells were obtained from age-matched donors with no prior history of ocular disease (controls) and from donors with confirmed glaucoma (Lion’s Eye Bank, Tampa, FL, USA and Novartis, Boston, MA, USA) within 24 h post-mortem. Glaucoma status was verified using available medical records and/or confirmation from family members. All tissue was obtained and handled in accordance with the Declaration of Helsinki.

The LC cells were isolated from donor optic nerves as described by Lopez et al. [18]. Cells were maintained in culture and passaged every 3 days using 0.25% trypsin/EDTA (Sigma, Dublin, Ireland), and all experiments were performed using early-passage cells (≤passage 8).

LC cell identity was established using characteristic morphology and previously described marker expression. LC cells demonstrate expression of collagen IV, laminin and αSMA, with absence of Factor VIII, GFAP and 3G5 expression, distinguishing them from endothelial cells, optic nerve head astrocytes and pericytes, respectively.

For single-cell RNA sequencing, LC cells from 2 normal and 3 glaucoma donors were analysed. Donors were age-matched, ranging from 79 to 88 years (controls) and 79 to 86 years (glaucoma), with mean ages of 85.8 and 83.6 years, respectively. For additional molecular and functional experiments, LC cells from a further 6 normal and 6 glaucoma donors were included. In total, including donors for IHC, LC cells from 10 normal and 13 glaucoma donors were used in this study (Supplementary Material S13). Detailed clinical metadata, including race/ethnicity and disease severity, were not consistently available for all donor tissues.

### 4.2. Single Cell RNA Sequencing (scRNA-Seq)

#### 4.2.1. Sample Preparation

Human LC cells from 2 normal and 3 glaucoma donors, all grown to confluency and used between passages 3 and 5, were subjected to scRNA-seq. Cells were detached by incubating with Papain/l-cysteine/EDTA mix (Worthington Biochemical, Lakewood, NJ, USA; Catalogue no. LK003150) for 30 min until complete detachment was seen in normal and glaucoma cell flasks [102]. Cell suspensions were then added to Eppendorf tubes with FBS and Ovomucoid protease inhibitor with bovine serum albumin (Fisher Scientific, Dublin, Ireland). Cell suspensions were centrifuged at 1000 rpm for 5 min. Supernatants were removed and cells resuspended in 1000 μL PBS/0.04% BSA solution (Fisher Scientific, Dublin, Ireland). Cell viability was assessed using trypan blue and calculated to be over 90% for all five suspensions. Cells were manually counted and diluted to a concentration of approximately 1000 cells/µL. Cell suspensions were pooled and then mixed by gently pipetting. Cell suspensions were processed on the 10× Genomics Chromium platform (10× Genomics, Pleasanton, CA, USA) at the Queen’s University Belfast (QUB) Genomics Core Technology Unit to capture a total of ~6000 cells. The scRNA-seq experiment was performed in two sequencing runs. In the first run, cell suspensions from two normal and two glaucoma donors were pooled and processed on the 10× Genomics Chromium platform. A third glaucoma donor was subsequently processed in a separate run.

#### 4.2.2. Single Cell Sequencing and Bioinformatic Analysis

scRNA-seq libraries were sequenced on an Illumina NextSeq 500 sequencer (Illumina, San Diego, CA, USA). Cell Ranger (version 6.0.1) was used to process raw sequencing data. Using cellranger mkfastq, BCL files were converted to FASTQ format, and then aligned to the human transcriptome (GRCh38) with cellranger count.

#### 4.2.3. Sample Demultiplexing

Single-cell count data from multiplexed cell suspensions were separated by individual genotype. Bulk RNA sequencing was performed on RNA extracted from cells of the respective individuals for genotyping. SNPs were called for each donor with bcftools ‘mpileup’ and ‘call’, with bcftools ‘filter’ applied to the resulting VCF data to retain variants with a quality score ≥ 20. Demuxlet software was then used to assign sample identity to each cell in the single-cell BAM files using the donor genotype [103]. For the multiplexed sequencing run, cells with genotype profiles consistent with contributions from more than one donor were identified as inter-donor doublets and excluded from downstream analysis. As the second sequencing run contained only a single donor, genotype-based identification of inter-donor doublets using Demuxlet was not applicable to this run. To assess within-donor doublets that could not be identified using genotype-based demultiplexing, DoubletFinder (v2.0.6) was applied separately to donors N1, N2, G2 and G3. Donor G1 was excluded because of insufficient cell numbers. Expected doublet rates were estimated using 10× Genomics guidance based on the number of recovered cells. Putative doublets were removed, and MAST differential-expression analysis was repeated to assess their influence on the reported findings.

#### 4.2.4. Data Processing and Dimensionality Reduction

Adjusted count matrices were analysed in R (4.2.2) using Seurat [104]. Samples were filtered to remove cells with fewer than 600 genes or with >20% counts mapped to mitochondrial genes resulting in 6131 cells passing QC, with median counts and genes per cell of 19,232 and 4204 respectively. Additional donor-level quality-control metrics, retained cell counts and the distribution of cells across the four transcriptional groups are provided in Supplementary Materials S14 and S15.

The top 2000 highly variable genes were scaled using ScaleData, regressing out cell–cell variation attributed to mitochondrial gene expression and total counts per cell. The scaled data were used as input for principal component (PC) analysis, from which the first 50 PCs were subjected to SNN clustering and UMAP generation. Clusters of cells were assigned to four broader transcriptional groups (Groups 1–4) based on proximity in UMAP space for marker-gene detection and differential-expression analysis. These groups were manually assigned and were not defined solely by selection of a particular unsupervised clustering resolution.

Cell-cycle scores were calculated and examined as part of quality-control analysis. SCTransform normalisation was repeated with cell-cycle effects regressed out, and the four broad transcriptional groups remained identifiable on the resulting UMAP (Supplementary Material S1). Quantitative comparison of group assignments before and after regression demonstrated that 98.57% of cells retained their corresponding group assignment. Fine-resolution clustering stability across different clustering resolutions was not assessed. Additionally, cells were annotated into predicted cell type using SingleR reference-based annotation, in which the expression profile of each cell was compared to the Human primary cell atlas [105,106].

#### 4.2.5. Differential Expression Analysis

Marker gene detection and differential expression testing was performed in Seurat using the MAST package [107]. Genes expressed in at least 10% of cells in either group being tested, with log2 fold-change > 0.25 and with adjusted *p*-value < 0.05, were considered significantly differentially expressed. Differential-expression testing was performed at the individual cell level. As an additional donor-level sensitivity analysis, pseudobulk differential-expression analysis was performed using DESeq2, with gene counts aggregated by individual donor. Donor G1 was excluded from this analysis because of the limited number of retained cells. Differential expression was assessed between glaucoma and normal donors, with adjusted *p*-values below 0.05 considered statistically significant. To assess the consistency of differential expression across sequencing runs, significantly altered genes identified by DESeq2 (adjusted *p* < 0.05) were further filtered to retain genes exhibiting concordant expression changes of at least twofold (|log2FC| > 1) in both glaucoma donors G2 and G3 relative to normal donors.

### 4.3. Gene Set Enrichment Analysis

Gene set enrichment analysis (GSEA) was conducted using the fgsea R package with default parameters and MSigDB Hallmark gene set [108]. Fold changes from the differential expression analysis step were used as input. The Hallmark gene set was downloaded using the msigdbr R package. Both raw sequence data and gene expression matrices have been submitted to the NCBI Gene Expression Omnibus database and will be publicly available.

### 4.4. Transcription Factor Analysis

Transcription factor activities were calculated using the Viper algorithm and a co-expression network generated [109]. To infer the network, the GENIE3 algorithm and gene expression profiles with fibroblast cell type selected data from the ARCHS4 database were used [40,41]. The ARCHS4 gene expression matrix with gene counts was log2-transformed and quantile-normalised. The i-cisTarget web tool with default parameters was used to calculate overrepresented TFs for the list of differentially expressed genes using PWM and TFBS ChIP databases [45,46].

### 4.5. Gel Contractility Assay

The collagen gel contraction assay was provided by Cell BioLabs, Inc., San Diego, CA, USA. LC cells were incubated in 24 well-plates and harvested and suspended in the appropriate culture medium at a density of 2–5 × 10^6^ cells/m, according to the manufacturer instructions. Collagen lattices were prepared by combining two parts of the cell suspension with eight parts of ice-cold collagen gel working solution. The mixture was gently triturated to ensure homogeneity while minimising the introduction of air bubbles. Control gels with no cells were included in parallel.

Aliquots of 0.5 mL of the cell–collagen mixture were dispensed into each well of a 24-well plate and incubated at 37 °C for 1 h to allow polymerisation. Following gelation, 1.0 mL of culture medium was carefully added on top of each collagen lattice.

Cultures were maintained for 48 h to permit the development of intrinsic contraction modulator 2,3-butanedione monoxime (BDM) within the gels. Cells were treated with [10 mM, BDM]) prior to gel release. Contraction was initiated by gently detaching the collagen gels from the well edges using a sterile spatula. Gel contraction was assessed at specified time points by measuring changes in gel diameter using a quantitative image analysis software, Image-Pro Plus Version 7.0.

### 4.6. RNA Isolation, cDNA Synthesis and Quantitative RT-qPCR

Based on the scRNA-seq results, some of the most dysregulated mRNAs were chosen for further validation by RT-qPCR in glaucomatous LC cells vs. healthy control LC cells, using different donors to those used for scRNA-seq. Total RNA extraction was performed using Tri-Reagent© protocol (Life Technologies, Dublin, Ireland), and cDNA synthesis was carried out using the oligo-dT primers, deoxy-nucleotides (dNTPs), and enhanced avian reverse transcriptase (eAMV) (Merck, Dublin, Ireland) cDNA, according to the manufacturer’s instructions. qPCR was performed using SYBR green master mix (Roche, Dublin, Ireland) on a LightCycler^®^ 480 System (ROCHE, Dublin, Ireland). Target gene expression levels were normalised to ribosomal 18S genes. Data are presented as expression levels relative to *18S gene* using the 2^−ΔCT^ method. Primer sequences are detailed in Supplementary Material S16.

### 4.7. Protein Extraction and Western Blotting

Total protein lysates were prepared using RIPA buffer containing protease/phosphatase inhibitor cocktail (Sigma/Merck, Arklow, Ireland) according to manufacturer’s instructions. Proteins were separated by sodium dodecyl sulphate-polyacrylamide gel electrophoresis (SDS-PAGE) followed by blotting on nitrocellulose membranes (Thermo Fisher Scientific, Dublin, Ireland). The following proteins were used: Antibodies against KRT18 (ab32118, Abcam, Cambridge, UK 1:2000), THBS1 (ab267397, Abcam, 1:1000), ATF4 (ab216839, Abcam, 1:1000), DDIT3 (ab11419, Abcam, 1:1000), HSPA5 (ab121390, Abcam, 1:1000), COL1A1 (ab316222, Abcam, 1:1000), ACTA2 (ab32575, Abcam, 1:1000), and β-Actin (ab8226, Abcam, 1:1000). HRP-conjugated anti-rabbit IgG H&L, (ab205718, Abcam, 1:10,000) and HRP-conjugated anti-mouse IgG (sc-516102, Santa Cruz Biotechnology, Dallas, TX, USA) were used as secondary antibodies for 1 h at room temperature. Protein signals were detected with Super Signal West Pico PLUS Chemiluminescent Substrate (Thermo Scientific, Athlone, Ireland) and analysed with ImageJ software (1.5x series, NIH, LOCI, University of Wisconsin, USA).

### 4.8. Immunofluorescence

LC cells were seeded at 1 × 10^4^/well and grown on Lab-Tek II 8-well chamber slides. Seventy-two hours post seeding, once the cells formed a confluent monolayer on the bottom of the chamber slide, the cells were fixed using 4% paraformaldehyde (pH 7.4) for twenty minutes at room temperature and washed three times in PBS for five minutes. Cell monolayers were blocked in PBS which contained 5% normal goat serum, 1% bovine serum albumin and 0.1% Triton X-100 at room temperature for one hour. Primary antibodies used were KRT18 (Abcam ab668), ATF4 (Abcam, ab184909) and p-EIF2α (Abcam ab184549), each diluted to the manufacturer’s recommendation. The primary antibody contained this blocking buffer and was incubated overnight at 4 °C, and then slides were washed 3 times for 5 min each in PBS. The secondary antibody was also made up in a blocking buffer (Abcam, 1:500) along with phalloidin (1:3000; Invitrogen, Carlsbad, CA, USA) and incubated for 2 h at room temperature in a humidity chamber. Slides were washed again 3 times for 5 min each in PBS. Following incubation, chamber slides were mounted with Aqua-Polymount (Polyscience, Warrington, PA, USA) after nuclei-counterstaining with DAPI at 1:5000 for 60 s. Fluorescent images of LC monolayers were captured using a confocal microscope (Zeiss LSM 710, Zeiss Microscopy, Oberkochen, Baden-Württemberg, Germany) and processed using imaging software ZEN 2012. ImageJ software was used to define regions of interest (ROIs) around individual cells, and corrected total cell fluorescence (CTCF) was calculated to quantify fluorescence intensity.

### 4.9. Immunohistochemistry

Paraffin-embedded formalin fixed posterior segments from glaucoma and aged matched control donor eyes (Wichita, Kansas; LEITR, Tampa; Portland, Oregon; UNTHSC, Texas; [Supplementary Material S17]) were progressively deparaffinised (100%, xylene, and 100%, 95%, 70% and 50% ethanol) and rinsed in phosphate-buffered saline (PBS 1X). Antigen retrieval was performed using a citrate buffer (pH 6) at approximately 70 °C for 90 min. Sections were stored overnight at 4 °C in a blocking buffer (10% goat serum/0.05% TX-100 in PBS). Sections were blocked (10% goat serum/2% TX-100 in PBS) at room temperature for 2 h. Tissue sections were incubated overnight at 4 °C with primary antibodies (GRP78, ab21685 (abcam), 1:100; CHOP, NBP2-13172 (Bio-Techne, Minneapolis, MN, USA), 1:100; anti-ATF4, 10835-1-AP (Thermo-Fisher Scientific), 1:100) diluted in blocking buffer (10% goat serum/2% TX-100 in PBS). After incubation, tissues were washed five times with PBS. Secondary antibody (Alexa Fluor 488 goat anti-rabbit, A11008, 1:500) was applied for 2 h to the sections at room temperature. After incubation, tissues were washed with PBS six times prior to being cover-slipped with Vectashield Plus antifade mounting medium with DAPI (Catalog No. H-2000-10; Vector Laboratories, Newark, CA, USA). Immunohistochemistry (IHC) imaging was performed using the Keyence microscope (BZ-X710; Keyence, Itasca, IL, USA) in a masked fashion. Images were captured with a 20X objective lens. To determine co-localisation of these three stress markers, GRP78, CHOP, and ATF4, with LC cells in ONH tissue, each stress marker was co-stained with α-smooth muscle actin (αSMA), since αSMA has been shown to label LC cells in situ [110] (Note: αSMA is not specific for LC fibroblasts and also labels vascular smooth-muscle cells/pericytes. αSMA staining was therefore interpreted in conjunction with anatomical localisation and cellular morphology, with LC cells identified lining or embedded within the laminar beams and vascular-associated αSMA-positive cells distinguished by their relationship to blood vessels). ONH tissue from the POAG donor (1057-10) with the highest staining for the stress markers was prepared as described above. This donor was selected to facilitate detection of both immunofluorescent signals in FFPE tissue. The co-localisation experiment was intended as a qualitative demonstration and was not used for quantitative comparison between glaucoma and control donors. The same 3 stress marker primary antibodies were used along with an anti-αSMA antibody (Invitrogen #14-9860-82, diluted 1:100). The primary αSMA antibody was detected using Alexa Fluor 568 goat anti-mouse IgG (H + L) (Invitrogen #A11031, diluted 1:500). As previously, the sections were counterstained with DAPI (blue) to visualise nuclei.

### 4.10. Treatment with ISRIB and 4-PBA

The Integrated Stress Response Inhibitor (ISRIB; SML0843) and the ER stress inhibitor 4-phenylbutyric acid (4-PBA; SML0309) were purchased from Sigma-Aldrich/Merck (Arklow, Ireland). The concentrations used for cellular treatments were guided by previously published studies and evaluated using an MTT assay to assess cell viability and potential cytotoxicity. ISRIB and 4-PBA were dissolved in dimethyl sulfoxide (DMSO) and used at final concentrations of 1 µM and 200 µM, respectively.

Normal LC (NLC) and glaucomatous LC (GLC) cells (*n* = 3 donors per group) were maintained under standard cell culture conditions (37 °C, 5% CO_2_, humidified atmosphere). Cells (1 × 10^6^) were seeded in T25 culture flasks. Once approximately 80% confluent, cells were serum-starved overnight prior to treatment. Cells were subsequently treated with ISRIB (1 µM) or 4-PBA (200 µM) for 24 h. Vehicle controls received 0.01% DMSO. RNA and/or protein were isolated from untreated control and ISRIB- or 4-PBA-treated cells using the RNA and protein isolation protocols described above.

The concentrations used for subsequent experiments were evaluated using an MTT assay (Sigma-Aldrich/Merck, Dublin, Ireland) to assess cell viability and potential dose-dependent cytotoxicity in NLC and GLC cells (*n* = 3 donors per group). Cells were exposed to varying concentrations of ISRIB (0.1, 1, 10 and 100 µM) or 4-PBA (10, 100, 200 and 1000 µM).

### 4.11. MTT Cell Viability Assay

Cell viability and the potential cytotoxic effects of ISRIB and 4-PBA on human LC cells were assessed using an MTT tetrazolium reduction assay (3-(4,5-dimethylthiazol-2-yl)-2,5-diphenyltetrazolium bromide; Sigma-Aldrich/Merck, Dublin, Ireland). MTT was prepared by dissolving the reagent in 10 mL phosphate-buffered saline (PBS; pH 7.4) and incubating overnight at 4 °C in the dark to ensure complete dissolution.

Normal and glaucomatous LC cells were seeded at a density of 1 × 10^4^ cells/well in 96-well plates. Cells were cultured for 48 h in serum-complete medium, followed by overnight incubation in serum-free medium. Cells were subsequently washed with PBS and assigned to control or treatment groups. Treatment groups were exposed to increasing concentrations of ISRIB (0.1, 1, 10 and 100 µM) or 4-PBA (10, 100, 200 and 1000 µM) for 24 h in 100 µL of medium containing 10% foetal bovine serum (FBS), while control wells received the corresponding vehicle control. Following treatment, 10 µL of MTT solution was added to each well, and plates were incubated for 4 h. Cell-free wells containing 100 µL of serum-complete medium served as negative controls. Following MTT incubation, 100 µL of isopropanol containing 0.04 N HCl was added to each well to solubilise the formazan crystals. Absorbance was measured using a SpectraMax^®^ Plus 384 microplate spectrophotometer with SoftMax^®^ Pro 6.2.2 software (Molecular Devices, San Jose, CA, USA) at 570 nm, with a reference wavelength of 630 nm. A single absorbance measurement was obtained following the 4 h MTT incubation period, in accordance with the manufacturer’s instructions. Cell viability was expressed as a percentage of the corresponding control. Experiments were performed using LC cells from 3 donors per group, with each donor analysed in triplicate.

### 4.12. Quantification of Cellular Metabolic Activity Following ISRIB Treatment

Cellular metabolic activity of cultured LC cells was assessed using the 3-(4,5-dimethylthiazol-2-yl)-5-(3-carboxymethoxyphenyl)-2-(4-sulphophenyl)-2H-tetrazolium (MTS) assay according to the manufacturer’s protocol. Cells (1 × 10^4^ cells/well; *n* = 3 donors per group) were plated in triplicate into 96-well plates in full-growth DMEM-F12 medium for 48 h. The medium was removed and cells were incubated overnight in serum-free medium. Cells were untreated (control) or treated with ISRIB at concentrations of 0.1 µM, 0.5 µM and 1.0 µM for different durations (6, 12, 24 and 48 h).

CellTiter^®^ 96 AQueous One Solution Reagent (20 µL; Promega, Dublin, Ireland) was added to each well at the time points of interest, followed by incubation for an additional 3 h. Following MTS incubation, absorbance was measured at 490 nm using a SpectraMax spectrophotometer (Molecular Devices Corp., Wokingham, UK), with a reference wavelength of 630 nm, and analysed using SoftMax Pro 7.1.1 software (Molecular Devices Corp., UK). Cellular metabolic activity was calculated relative to the untreated control using the formula: (OD_ISRIB − OD_Blank)/(OD_Control − OD_Blank). Technical replicate measurements from each donor were averaged prior to statistical analysis.

### 4.13. Statistical Analysis

Experimental data are presented as mean ± standard deviation (SD) or standard error of the mean (SEM), as indicated in the relevant figure legends. Statistical analyses were performed using the individual eye donor as the biological replicate rather than treating technical replicates as independent observations. Where multiple technical measurements were obtained from cells derived from the same donor, these measurements were first averaged to generate a single donor-level value prior to statistical analysis. Comparisons between two independent groups were performed using a two-tailed unpaired Student’s *t*-test, where appropriate. Where control and treatment conditions were derived from the same donor, comparisons were analysed as paired samples. For comparisons involving more than two groups, one-way analysis of variance (ANOVA) followed by Tukey–Kramer post hoc testing was applied. Thus, the reported *n* represents the number of independent eye donors included in each experimental group. Statistical significance was set at *p* < 0.05.

## 5. Conclusions

In this study, we characterised transcriptional changes in human lamina cribrosa cells from normal and glaucomatous eyes, and identified signalling pathways contributing to optic nerve head remodelling. Previously, we have demonstrated increased ECM gene expression, fibrosis, cell proliferation, oxidative stress/mitochondrial dysfunction and abnormal bioenergetics (reduced oxidative phosphorylation and increased glycolysis) in glaucoma LC cells [17,76,111,112]. In the current study, our findings support a fibroblast/contractile myofibroblast phenotype in cultured glaucomatous LC cells, characterised by increased ECM gene expression and altered cell-cycle-associated transcriptional programmes. Our findings also support a potential association between ISR/ER-stress-associated signalling and the glaucomatous LC phenotype, including altered expression of ECM-related genes. Pharmacological modulation with ISRIB and 4-PBA was associated with reduced expression of several stress-response and ECM-related genes. These findings support further investigation of ISR/ER-stress-associated pathways and their pharmacological modulation in LC remodelling, but do not establish a direct causal relationship between ISR activation, LC fibrosis and ONH cupping.

## Figures and Tables

**Table 1:** **Master regulons identified by VIPER analysis.**

Regulon	Size	NES	*p* Value	FDR	Ledge (Genes)
HMGB1	93	5.47	4.58 × 10^−8^	8.71 × 10^−7^	H2AFZ, STMN1, SSBP1, +63 genes
CENPA	66	3.69	2.26 × 10^−4^	2.15 × 10^−3^	TUBA1B, H2AFZ, STMN1, +62 genes
HMGB2	49	3.09	2.00 × 10^−3^	9.48 × 10^−3^	STMN1, PTTG1, TYMS, +42 genes
SSRP1	67	2.91	3.57 × 10^−3^	1.36 × 10^−2^	TUBA1B, TUBA1C, EIF5A +58 genes
TCF19	29	2.49	1.28 × 10^−2^	4.05 × 10^−2^	TUBA1B, TUBB4B, TUBA1C, +26 genes
SUB1	62	2.34	1.94 × 10^−2^	5.27 × 10^−2^	MGST1, SSBP1, POMP, +36 genes
WDHD1	33	1.91	5.67 × 10^−2^	1.35 × 10^−1^	RRM2, TOP2A, MAD2L1, +27 genes
TAX1BP1	67	1.53	1.27 × 10^−1^	2.42 × 10^−1^	MGST1, MYL12B, RPL22L1, + 9 genes
CSDE1	26	−1.66	9.63 × 10^−2^	2.03 × 10^−1^	MLEC, EIF3A, STT3B, +14 genes
TCF25	45	−3.30	9.65 × 10^−4^	6.11 × 10^−3^	PLEKHO1, MAF1, SLC39A7, +28 genes

Table of master regulons identified by VIPER analysis. Regulons with an absolute normalised enrichment score (NES) > 1.5 are shown. Ledge = leading-edge analysis. FDR = false discovery rate.

## Data Availability

The single-cell RNA-sequencing datasets generated during this study have been deposited in the NCBI Gene Expression Omnibus (GEO) under accession number GSE351018. Additional data supporting the findings of this study are available from the corresponding author upon reasonable request.

## Supplementary Materials

The following supporting information can be downloaded at: https://www.mdpi.com/article/10.3390/ijms27219892/s1.
