# Supplementary material for: Single-Cell RNA Sequencing of Human Lamina Cribrosa Fibroblasts in Glaucoma
Source: Int J Mol Sci. 2026 Nov 4;27(21):9892. doi: 10.3390/ijms27219892 (PMC13764452; doi:10.3390/ijms27219892)
Supplement: Supplementary file 1 [file ijms-27-09892-s001.zip › ijms-4525317-supplementary.pdf]

Supplementary Materials

Supplementary Material S1. UMAP visualisation following cell-cycle regression.

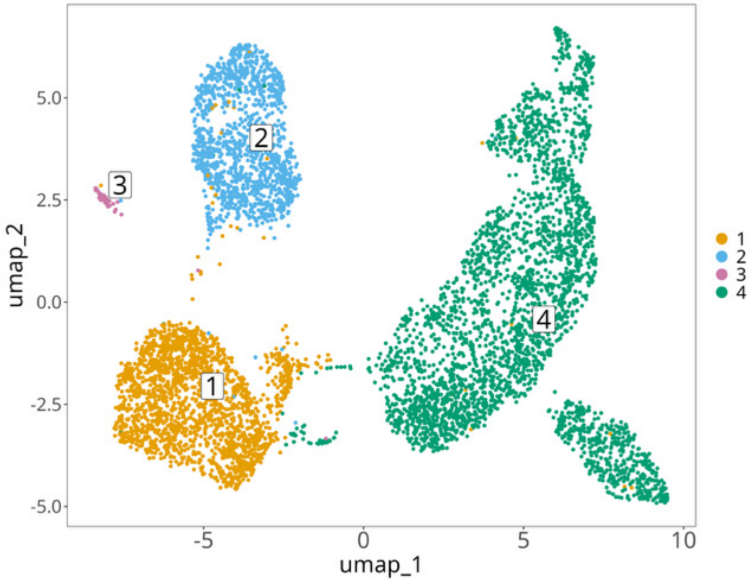

Supplementary Material S2. Cell cycle regression group-assignment concordance matrix.

	**Post-CC Groups**			
**Original Groups**	**1**	**2**	**3**	**4**
**1**	98.45%	1.01%	0.05%	0.48%
**2**	0.38%	99.54%	0	0.08%
**3**	3.12%	0	96.88%	0
**4**	1.63%	0.1%	0	98.28%

Supplementary Material S3. Gene expression across all four groups.

https://docs.google.com/spreadsheets/d/1tJbsVf-QAxB5_P-cBEY8j3WxEKTnSwPU/edit?usp=drive_link&ouid=112747493113399009415&rtpof=true&sd=true

Supplementary Material S4. Differential over expressed genes across all four groups.

https://docs.google.com/spreadsheets/d/1VlnWyP9fpQzYHoXqc5FS5J8ZZFtVpfiy/edit?usp=drive_link&ouid=112747493113399009415&rtpof=true&sd=true

Supplementary Material S5. Normal versus glaucoma differential gene expression.

https://docs.google.com/spreadsheets/d/1NdSPhOdC6mnD3KWPknp16-X0K5gNuLnR/edit?gid=192290764#gid=192290764

Supplementary Material S6. Donor-level pseudobulk differential-expression analysis using DESeq2.

https://docs.google.com/spreadsheets/d/1SFsSLXTE4iTw2Qa_UyE4N0yD9q2idEqI/edit?usp=drive_link&ouid=112747493113399009415&rtpof=true&sd=true

Supplementary Material S7. 688 robust donor concordant genes

https://docs.google.com/spreadsheets/d/1XOJzNzZRSgCry4NwyXUDxbFeIPRE0Hkz/edit?usp=drive_link&ouid=112747493113399009415&rtpof=true&sd=true

Supplementary Material S8. Pathway enrichment analysis following exclusion of glaucoma donor G3. (A) Enrichment of genes showing concordant differential expression with the original analysis, including ECM, fibroblast, TGF-β, hypoxia and EMT-associated pathways. (B) Enrichment of 1,146 newly identified differentially expressed genes, highlighting preservation of ECM, EMT-associated and TGF-β signalling pathways, alongside laminin, integrin and reelin signalling.

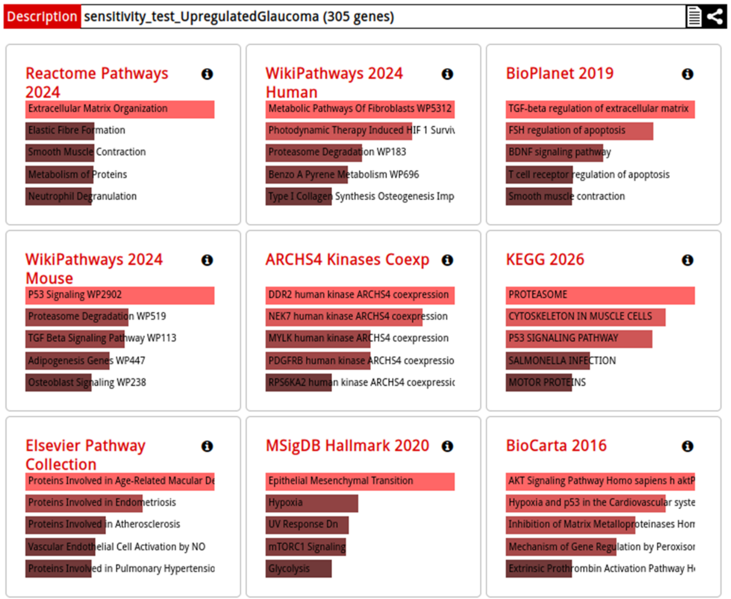

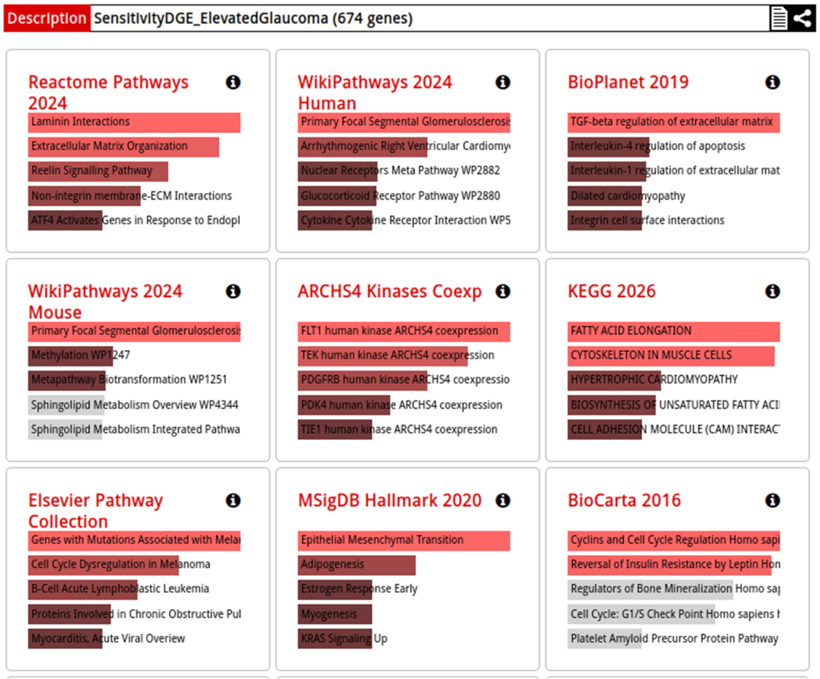

Supplementary Material S9. Within-donor doublet detection and sensitivity analysis

	Singlet	Doublet
N1	2304	114
N2	1197	29
G2	146	1
G3	2470	51

DoubletFinder v2.0.6 was applied separately to individual donors, excluding G1 because of insufficient cell numbers. Repeating the MAST differential-expression analysis after removal of predicted within-donor doublets demonstrated near-identical differential-expression estimates compared with the original analysis (Spearman's r = 0.999).

Supplementary Material S10. Gene list for each regulon identified by Viper analysis.

https://docs.google.com/spreadsheets/d/1PrTfTreXaBvJLJzKvI_PNoDy3NzVSicU/edit?usp=drive_link&ouid=112747493113399009415&rtpof=true&sd=true

Supplementary Material S11. i-cisTarget results.

https://drive.google.com/file/d/1BR7JJKjBhXuxJPKf-c-xwuXRaLy3Vn_V/view?usp=drive_link

Supplementary Material S12. String database connections between genes of interest.

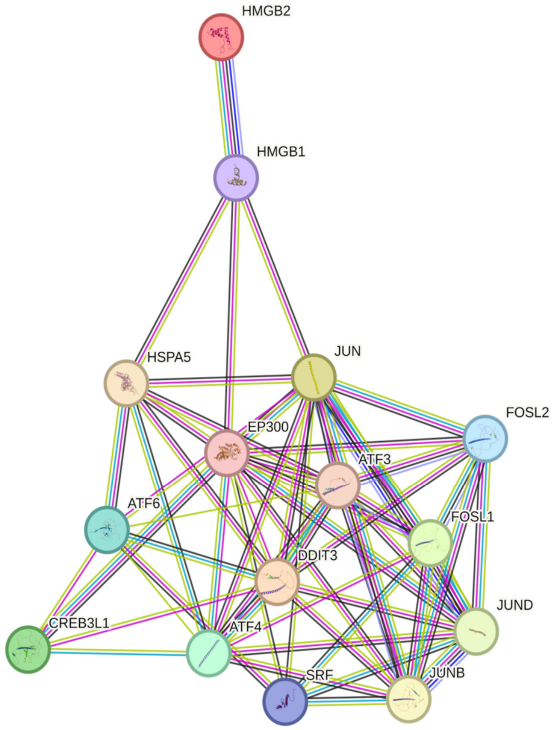

Supplementary Material S13. Donor details for cell culture

Donor ID	Age	Gender	Disease State	Eye Bank
276-06	86	M	None	Cambridge, MA (Novartis)
438-07	84	F	Glaucoma	Cambridge, MA (Novartis)
1325-06	79	F	None	Cambridge, MA (Novartis)
644-03	84	M	Glaucoma	Cambridge, MA (Novartis)
428-07	83	M	Glaucoma	Cambridge, MA (Novartis)
82-02	88	F	None	Central Florida (Tampa)
439-02	79	M	None	Central Florida (Tampa)
444-02	87	M	None	Central Florida (Tampa)
553-02	87	F	None	Central Florida (Tampa)

**350-02**	**84**	**F**	**Glaucoma**	**Central Florida (Tampa)**
**412-02**	**85**	**M**	**Glaucoma**	**Central Florida (Tampa)**
**600-02**	**86**	**M**	**Glaucoma**	**Central Florida (Tampa)**
**652-02**	**79**	**M**	**Glaucoma**	**Central Florida (Tampa)**

Table- donor details including age, gender, condition, source.

Supplementary Material S14. Single-cell RNA-sequencing quality-control UMAPs.

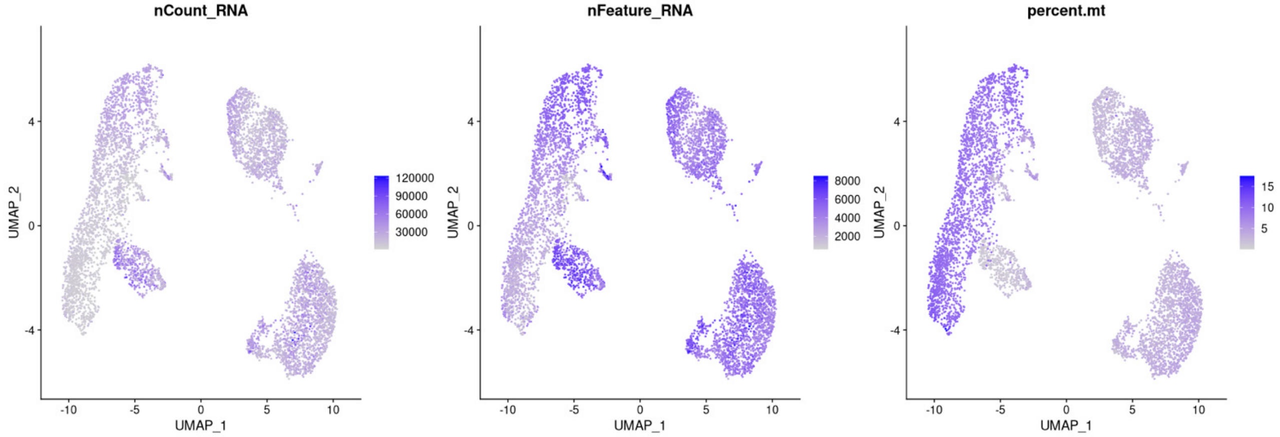

Supplementary Material S15. Donor-level quality-control metrics and transcriptional group composition. Summary of retained cell counts, gene and transcript counts, mitochondrial and ribosomal transcript percentages for each donor, together with the distribution of donor-derived cells across Groups 1–4. https://docs.google.com/spreadsheets/d/1MjF7AabmLoLm-samJjxDMPsxFzf932YG/edit?usp=drive_link&ouid=112747493113399009415&rtpof=true&sd=true

Supplementary Material S16. Primers used for RT-qPCR

**Human Gene Symbol**	**Forward primers**	**Reverse primers**
KRT18	ATCTTGGTGATGCCTTGGAC	CTGCTGGGGGCTGAGGTCT
THBS1	GACAGGGTCTACGGGACGTGTTTC	TGCAGAGGTAGTTATAAAGGCAG

E2F1	GGATCTGGAGACTGACCATCAG	GGTTTCATAGCGTGACTTCTCCC
RAD54L	TTTACGCCAGAGTCCAGAGTG	ATGAAGGCGGAAGGTCTCATA
MYC	CCTGGTGCTCCATGAGGAGAC	CAGACTCTGACCTTTTGCCAGG
SNAI1	TGCCCTCAAGATGCACATCCGA	GGGACAGGAGAAGGGCTTCTC
ATF4	ATGGCCGGCTATGGATGAT	CGAAGTCAAACTCTTTCAGATCCATT
DDIT3	CATGAACAGTGGGCATCACC	GCTGGGTACACTTCCGGAGAG
HSPA5	CACGTCCAACCCCGAGAA	ATTCCAAGTGCGTCCGATG
ACTA2	CCGACCGAATGCAGAAGGA	ACAGAGTATTTGCGCTCCGAA
COL1A1	ACGAAGACATCCCACCAATC	ATGGTACCTGAGGCCGTTC
FN1	ACAACACCGAGGTGACTGAGAC	GGACACAACGATGCTTCCTGAG
Ribosomal 18S	TGTGCCGCTAGAGGTGAAATT	TGGCAAATGCTTTCGCTTT

Supplementary Material S17. Donor details for immunostaining of human ONH tissues

Donor ID	Age	Gender	Disease State	Eye Bank
**115-11**	**81**	**M**	**Glaucoma**	**Wichita, KS**
**600-10**	**80**	**F**	**Glaucoma**	**LEITR (Tampa)**
**608-10**	**76**	**M**	**Glaucoma**	**Wichita, KS**
**1057-10**	**83**	**F**	**Glaucoma**	**Portland, OR**

**93651**	**60**	**F**	**Glaucoma**	**UNTHSC WBP**
**100810**	**60**	**F**	**Glaucoma**	**UNTHSC WBP**
**89467**	**73**	**M**	**Normal**	**UNTHSC WBP**
**90695**	**75**	**F**	**Normal**	**UNTHSC WBP**
**99566**	**79**	**F**	**Normal**	**UNTHSC WBP**
**100806**	**41**	**F**	**Normal**	**UNTHSC WBP**

Table- donor details including age, gender, condition, source.
